# The Association Between Genetically Predicted Systemic Inflammatory Regulators and Polycystic Ovary Syndrome: A Mendelian Randomization Study

**DOI:** 10.3389/fendo.2021.731569

**Published:** 2021-09-27

**Authors:** Hanxiao Chen, Yaoyao Zhang, Shangwei Li, Yuanzhi Tao, Rui Gao, Wenming Xu, Yihong Yang, Kemin Cheng, Yan Wang, Lang Qin

**Affiliations:** ^1^ Reproductive Centre, Department of Obstetrics and Gynaecology, West China Second University Hospital, Sichuan University, Chengdu, China; ^2^ West China School of Medicine, Sichuan University, Chengdu, China; ^3^ Key Laboratory of Birth Defects and Related Diseases of Women and Children of the Ministry of Education, West China Second University Hospital, Sichuan University, Chengdu, China; ^4^ Sichuan University-The Chinese University of Hong Kong (SCU–CUHK) Joint Laboratory for Reproductive Medicine, West China Second University Hospital, Sichuan University, Chengdu, China; ^5^ Outpatient Department, West China Second University Hospital, Sichuan University, Chengdu, China; ^6^ Department of Obstetrics and Gynaecology, Sichuan Academy of Medical Sciences & Sichuan Provincial People’s Hospital, Chengdu, China

**Keywords:** polycystic ovary syndrome, inflammation, cytokine, growth factor, Mendelian randomization

## Abstract

Polycystic ovary syndrome (PCOS) is one of the most common endocrine and metabolic diseases among women of reproductive age. Inflammation may be involved in the pathogenesis of PCOS, but its exact relationship with PCOS remains unclear. Herein, we investigate the causal association between systemic inflammatory regulators and PCOS risk through a two-sample Mendelian randomization (MR) approach based on the latest and largest genome-wide association study (GWAS) of 41 systemic inflammatory regulators in 8293 Finnish participants and a GWAS meta-analysis consisting of 10,074 PCOS cases and 103,164 controls of European ancestry. Our results suggest that higher levels of IL-17 and SDF1a, as well as lower levels of SCGFb and IL-4, are associated with an increased risk of PCOS (OR = 1.794, 95% CI = 1.150 – 2.801, P = 0.010; OR = 1.563, 95% CI = 1.055 – 2.315, P = 0.026; OR = 0.838, 95% CI = 0.712 – 0.986, P = 0.034; and OR = 0.637, 95% CI = 0.413 – 0.983, P = 0.042, respectively). In addition, genetically predicted PCOS is related to increased levels of IL-2 and VEGF (OR = 1.257, 95% CI = 1.022 – 1.546, P = 0.030 and OR = 1.112, 95% CI = 1.006 – 1.229, P = 0.038, respectively). Our results indicate the essential role of cytokines in the pathogenesis of PCOS. Further studies are warranted to assess the possibility of these biomarkers as targets for PCOS prevention and treatment.

## Introduction

Polycystic ovary syndrome (PCOS) is the most common endocrine and metabolic disorder among women of reproductive age (1, 2). PCOS is a complex and multifaceted disorder that is often diagnosed based on hyperandrogenism, ovulatory dysfunction and polycystic ovaries (3). Newer diagnostic tools include serum anti-Müllerian hormone (AMH) (4), among others. Although PCOS can lead to many complications, including obesity, infertility, metabolic disorders and cardiovascular disease, and although it has a significant impact on the patient’s quality of life (3), the exact cause of PCOS remains uncertain, and current treatments for PCOS mainly target its symptoms. Many studies have investigated potential risk factors for PCOS, which include genetic factors, excess androgen and insulin resistance (IR) (3). In addition, several other risk factors have been identified, such as obesity, chronic disease and low-grade inflammation (2, 5). Regarding inflammation, observational, epidemiological studies have demonstrated that circulating levels of several cytokines may be involved in the pathogenesis of PCOS. Some studies have explored the pathophysiological roles of cytokines, such as interleukin-1β (IL-1β), interleukin-1 receptor antagonist (IL1Ra), interleukin-6 (IL-6), interleukin-17 (IL-17) and interleukin-18 (IL-18) in PCOS development (6, 7). The association of the pathogenesis of PCOS with growth factors, such as vascular endothelial growth factor (VEGF), fibroblast growth factor (FGF) and pigment epithelium-derived factor (PEDF) has also been investigated by previous studies (8–10). Previous studies have also found that anti-inflammatory therapy can benefit patients with PCOS (11).

These associations are, however, derived from conventional observational studies and are thus susceptible to biases, such as small sample size, reverse causation and potential confounders (12). Based on the results of previous studies, it is sometimes difficult to reach a convincing conclusion because of these biases and the inevitable heterogeneity between different studies. Therefore, the causal effect of individual cytokines on the risk of PCOS remains uncertain. A Mendelian randomization (MR) study is less likely to be affected by reverse causality and potential environmental-social confounding factors because it uses genetic variants that are strongly and solely associated with exposure as instrumental variables (IVs) to establish causal association.

Hence, in the present study, we implemented a two-sample MR to investigate certain systemic inflammatory regulators as potential contributors to the pathogenesis of PCOS. In addition, the association between genetically predicted PCOS and systemic inflammatory regulators was also evaluated, offering a clear picture of the relationship between PCOS and inflammatory markers.

## Materials and Methods

### The Genetic Association Between Systemic Inflammatory Regulators and PCOS

Single nucleotide polymorphisms (SNPs) associated with cytokines and other systemic inflammatory regulators were obtained and selected from the summary statistics of the latest and largest genome-wide association study (GWAS) of 41 systemic inflammatory regulators in 8293 Finnish participants from three cohort studies, including the Cardiovascular Risk in Young Finns Study, the FINRISK1997 study and the FINRISK200225 study (13). Genetic associations were adjusted for age, sex, body mass index and the first ten genetic principal components. Genetic association data on PCOS in Europeans were selected from a GWAS meta-analysis of PCOS consisting of 10,074 PCOS cases and 103,164 controls of European ancestry (14). These genetic associations were adjusted for age in the cohort.

### The Selection of Genetic Instruments

For each of the 41 cytokines and other systemic inflammatory regulators (**Supplementary Table S1**), we extracted the SNPs that strongly predicted exposures at the genome-wide significance level (P < 5 × 10^–8^). To avoid potential pleiotropy, SNPs associated with more than one systemic inflammatory regulator were removed. Linkage disequilibrium (LD) was checked for using the European 1000 G reference panel as a reference, and SNPs with r^2^ < 0.1 were selected to omit the superposition effect of correlated SNPs. After harmonizing the selected SNPs with PCOS GWAS data, only nine systemic inflammatory regulators had more than two independent SNPs at a genome-wide significance level (**Supplementary Table S1**). Thus, an alternative cut-off (P < 5 × 10^–6^) was adopted to obtain SNPs predicting systemic inflammatory regulators. Eventually, all 41 systemic inflammatory regulators were selected under this condition (**Supplementary Table S2**). To avoid weak IVs, average SNP-specific F-statistics were calculated (15, 16), and IVs with F-statistics > 10 were considered as strong IVs for MR analysis.

For selecting IVs of PCOS, LD (r^2^ < 0.01) and palindromes were tested for the 14 SNPs estimated to be correlated to PCOS at the genome-wide significance level (P < 5×10^-8^). Eventually, after harmonizing the selected SNPs with systemic inflammatory regulators GWAS data, ten SNPs in total were included to construct the genetic IVs for PCOS (**Supplementary Table S3**).

### The Expression Level of Associated Systematic Inflammatory Regulators in Clinical Participants

In this study, we enrolled patients diagnosed with PCOS and age-matched healthy women in Reproductive Center, Department of obstetrics and gynecology, West China Second University Hospital from January, 2021 to August, 2021. PCOS was diagnosed according to the European Society for Human Reproduction and Embryology/American Society for Reproductive Medicine (ESHRE/ASRM) (Rotterdam criteria). Blood sample of each participant was collected and tested for the associated systematic inflammatory regulators using enzyme-linked immunosorbent assay (ELISA) kit (Shanghai Enzyme-linked Biotechnology Co., Ltd.). This study was approved by the Ethical Review Board of West China Second University Hospital, Sichuan University.

### Statistical Analysis

To obtain a reliable foundation for MR analysis, three prerequisite assumptions must be satisfied (17): (i) the IVs are correlated with exposure; (ii) the IVs affect the outcome only through their effects on exposure; and (iii) the IVs are independent of any confounders for the association between exposure and outcome. The inverse-variance weighted (IVW) method with random effects was used as the primary MR analysis to test the association between systemic inflammatory regulators levels and PCOS. Odds ratios (OR) and 95% confidence intervals (CIs) for PCOS were estimated, and a P < 0.05 was considered as statistically significant. Regarding sensitivity analyses, MR-Egger regression, weighted median (18), simple mode (19) and weighted mode methods were applied. The MR-Egger regression aimed to test the potential pleiotropic bias (20). We also applied the MR-Pleiotropy Residual Sum and Outlier (MR-PRESSO) method to detect and correct the horizontal pleiotropy and potential outliers (21). Heterogeneity was tested for by applying Cochran’s Q test on the IVW and MR-Egger estimates. Moreover, we performed bidirectional MR analysis to test the association between genetically predicted PCOS and systemic inflammatory regulators. MR analyses and sensitivity analyses were performed in R (version 4.0.2) using the TwoSampleMR package (version 0.5.5) and the MRPRESSO package (version 1.0). For laboratory data, continuous variables were expressed as mean ± standard deviation (SD) and student’s t-test was adopted for the comparison between the two groups. Binary logistic regression analyses were used to determine the ORs and the corresponding 95% CIs. For all comparisons, a two-sided p value < 0.05 was considered statistically significant. And statistical analyses were performed using SPSS version 22.0 (IBM, Armonk, NY, USA).

## Results

### The Association Between Genetically Predicted Systemic Inflammatory Regulators and PCOS

Among the 41 systemic inflammatory regulators, 17 had at least one genome-wide significant SNP, whereas all 41 had at least one SNP when using the higher cut-off (P < 5 × 10^–6^). All these SNPs were included in the analyses. All F-statistics were above 10, indicating that the results were less likely to be affected by the weak instruments bias.

**Figure 1:**
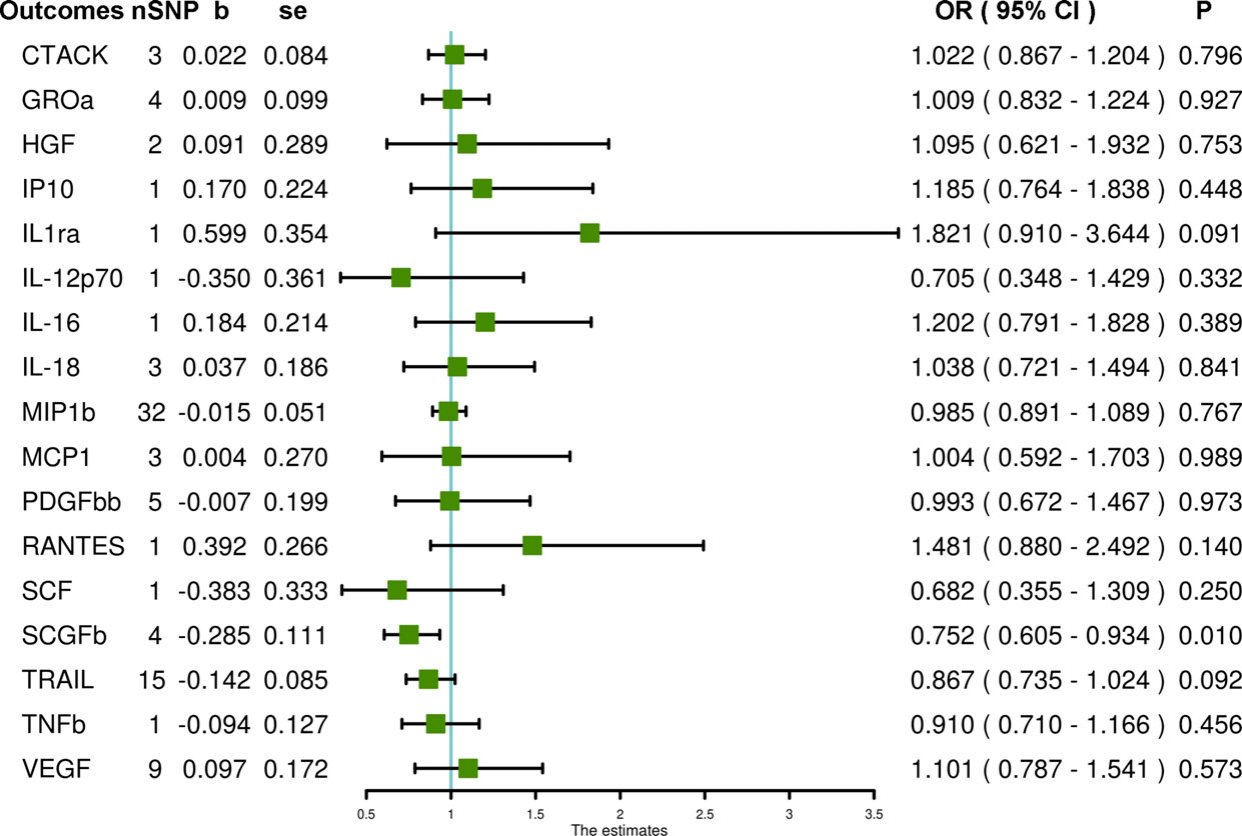
The association of systemic inflammatory regulators with PCOS using SNPs at the genome-wide significance level (P < 5 × 10^–8^): the inverse-variance weighted (IVW) method was applied as the primary method for MR analysis. SNP, single nucleotide polymorphism; b, beta coefficient; se, standard error; OR, odds ratio; CI, confidence interval; MR-PRESSO, Mendelian randomization pleiotropy residual sum and outlier; CTACK, cutaneous T-cell attracting chemokine; GROa, growth-regulated protein alpha; HGF, hepatocyte growth factor; IP10, interferon gamma-induced protein 10; IL1ra, interleukin-1-receptor antagonist; IL-12p70, interleukin-12p70; IL-16, interleukin-16; IL-18, interleukin-18; MIP1b, macrophage inflammatory protein 1b; MCP1, monocyte chemoattractant protein-1; PDGFbb, platelet-derived growth factor BB; RANTES, regulated on activation, normal T cell expressed and secreted; SCF, stem cell factor; SCGFb, stem cell growth factor beta; TRAIL, TNF-related apoptosis-inducing ligand; TNFb, tumor necrosis factor beta; VEGF, vascular endothelial growth factor.

In the primary MR IVW analyses using 17 systemic inflammatory regulators with SNPs that strongly predicted exposures at the genome-wide significance level (P < 5 × 10–8), our results show that only the stem cell growth factor beta (SCGFb) level is inversely associated with PCOS (OR = 0.752, 95% CI = 0.605 – 0.934, P = 0.010). MR-Egger and MR-PRESSO do not detect potential horizontal pleiotropy for SCGFb (P = 0.516 and P = 0.926, respectively). Heterogeneity is not detected for SCGFb (Cochran P value = 0.838). In the MR analyses using the weighted median method, the SCGFb level remains inversely associated with PCOS (OR = 0.747, 95% CI = 0.576 – 0.970, P = 0.029), and the TNF-related apoptosis-inducing ligand (TRAIL) level is also inversely associated with PCOS (OR = 0.828, 95% CI = 0.708 – 0.968, P = 0.018). Also, other systemic inflammatory regulators, including cutaneous T-cell attracting chemokine (CTACK), growth-regulated protein alpha (GROa), hepatocyte growth factor (HGF), interleukin-12p70 (IL-12p70), interleukin-16 (IL-16), IL-18, IL1ra, interferon gamma-induced protein 10 (IP10), monocyte chemoattractant protein-1 (MCP1), macrophage inflammatory protein 1b (MIP1b), platelet-derived growth factor BB (PDGFbb), regulated on activation, normal T cell expressed and secreted (RANTES), stem cell factor (SCF), tumor necrosis factor beta (TNFb) and VEGF are not significantly associated with PCOS in any analyses (**Figure 1** and **Supplementary Table S4**).

**Figure 2:**
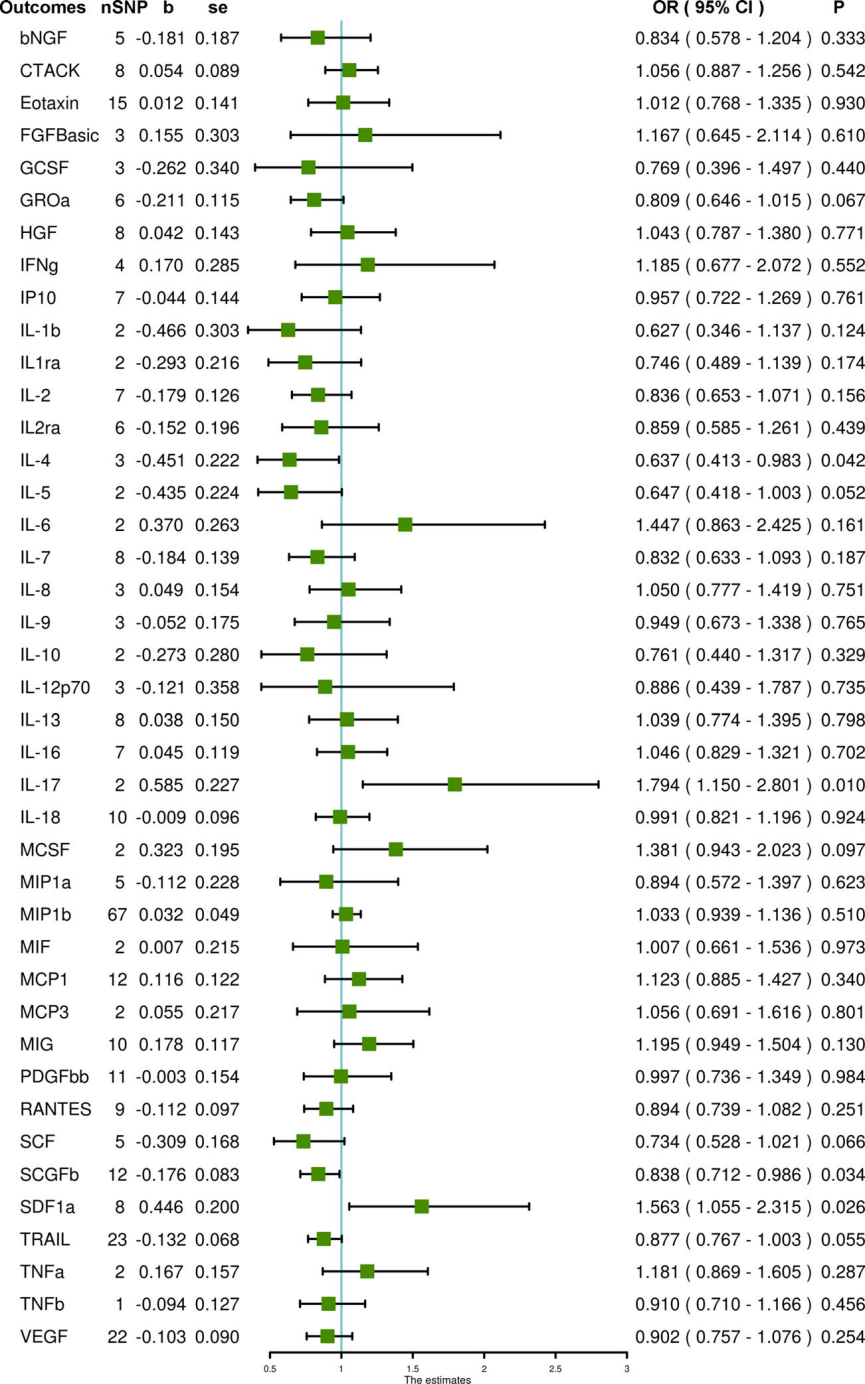
The association of systemic inflammatory regulators with PCOS using SNPs reaching the P < 5 × 10^–6^ significance level: the inverse-variance weighted (IVW) method was applied as the primary method for MR analysis. SNP, single nucleotide polymorphism; b, beta coefficient; se, standard error; OR, odds ratio; CI, confidence interval; MR-PRESSO, Mendelian randomization pleiotropy residual sum and outlier; bNGF, beta-nerve growth factor; CTACK, cutaneous T-cell attracting chemokine; FGFBasic, fibroblast growth factor basic; GCSF, granulocyte-colony stimulating factor; GROa, growth-regulated protein alpha; HGF, hepatocyte growth factor; IFNg, interferon gamma; IP10, interferon gamma-induced protein 10; IL-1b, interleukin-1-beta; IL1ra, interleukin-1-receptor antagonist; IL-2, interleukin-2; IL2ra, interleukin-2 receptor antagonist; IL-4, interleukin-4; IL-5, interleukin-5; IL-6, interleukin-6; IL-7, interleukin-7; IL-8, interleukin-8; IL-9, interleukin-9; IL-10, interleukin-10; IL-12p70, interleukin-12p70; IL-13, interleukin-13; IL-16, interleukin-16; IL-17, interleukin-17; IL-18, interleukin-18; MCSF, macrophage colony stimulating factor; MIP1a, macrophage inflammatory protein 1a; MIP1b, macrophage inflammatory protein 1b; MIF, macrophage migration inhibitory factor; MCP1, monocyte chemoattractant protein-1; MCP3, monocyte chemoattractant protein-3; MIG, monokine induced by gamma interferon; PDGFbb, platelet-derived growth factor BB; RANTES, regulated on activation, normal T cell expressed and secreted; SCF, stem cell factor; SCGFb, stem cell growth factor beta; SDF1a, stromal-cell-derived factor 1 alpha; TRAIL, TNF-related apoptosis-inducing ligand; TNFa, tumor necrosis factor alpha; TNFb, tumor necrosis factor beta; VEGF, vascular endothelial growth factor.

When using SNPs with a higher cut-off of significance P < 5 × 10^–6^, the SCGFb level and interleukin-4 (IL-4) level are inversely associated with PCOS (OR = 0.838, 95% CI = 0.712 – 0.986, P = 0.034 and OR = 0.637, 95% CI = 0.413 – 0.983, P = 0.042, respectively), whereas IL-17 and stromal-cell-derived factor 1 alpha (SDF1a) levels are associated with an increased risk of PCOS (OR = 1.794, 95% CI = 1.150 – 2.801, P = 0.010 and OR = 1.563, 95% CI = 1.055 – 2.315, P = 0.026, respectively) using IVW methods. MR-Egger and MR-PRESSO do not detect potential horizontal pleiotropy for SCGFb (P = 0.245 and P = 0.461, respectively), SDF1a (P = 0.105 and P = 0.055, respectively), IL-4 (P = 0.624 and P = 0.509, respectively) and IL-17 (PMR-PRESSO = 0.968). Also, heterogeneity is not detected for SCGFb (Cochran P value = 0.404), SDF1a (Cochran P value = 0.175), IL-4 (Cochran P value = 0.657) and IL-17 (Cochran P value = 0.983). In MR analyses using the weighted median method, the SCGFb level remains negatively associated with PCOS (OR = 1.879, 95% CI = 1.182 – 2.987, P = 0.008); the SDF1a level remains positively associated with PCOS (OR = 0.768, 95% CI = 0.617 – 0.956, P = 0.018), but the IL-4 level is not significantly associated with PCOS (OR = 0.722, 95% CI = 0.419 – 1.243, P = 0.240). The weighted median method is not suitable for IL-17 due to the limited number of SNPs. In addition, using the weighted median method, the TRAIL level is negatively associated with PCOS (OR = 0.832, 95% CI = 0.711 – 0.974, P = 0.021), and the monokine induced by the gamma interferon (MIG) level is positively associated with PCOS (OR = 1.293, 95% CI = 1.012 – 1.652, P = 0.040). Also, using the simple mode method, the macrophage inflammatory protein 1b (MIP1b) level is inversely related to PCOS (OR = 0.714, 95% CI = 0.517 – 0.986, P = 0.045). Other systemic inflammatory regulators, including beta-nerve growth factor (bNGF), Eotaxin, fibroblast growth factor basic (FGFBasic), granulocyte-colony stimulating factor (GCSF), interferon gamma (IFNg), interleukin-1-beta (IL-1b), interleukin-2 (IL-2), interleukin-2 receptor antagonist (IL2ra), interleukin-5 (IL-5), interleukin-6 (IL-6), interleukin-7 (IL-7), interleukin-8 (IL-8), interleukin-9 (IL-9), interleukin-10 (IL-10), interleukin-13 (IL-13), macrophage colony stimulating factor (MCSF), macrophage inflammatory protein 1a (MIP1a), macrophage migration inhibitory factor (MIF), monocyte chemoattractant protein-3 (MCP3) and tumor necrosis factor alpha (TNFa) are not associated with PCOS in any analyses (**Figure 2** and **Supplementary Table S5**).

### The Association Between Genetically Predicted PCOS and Systemic Inflammatory Regulator Levels

**Figure 3:**
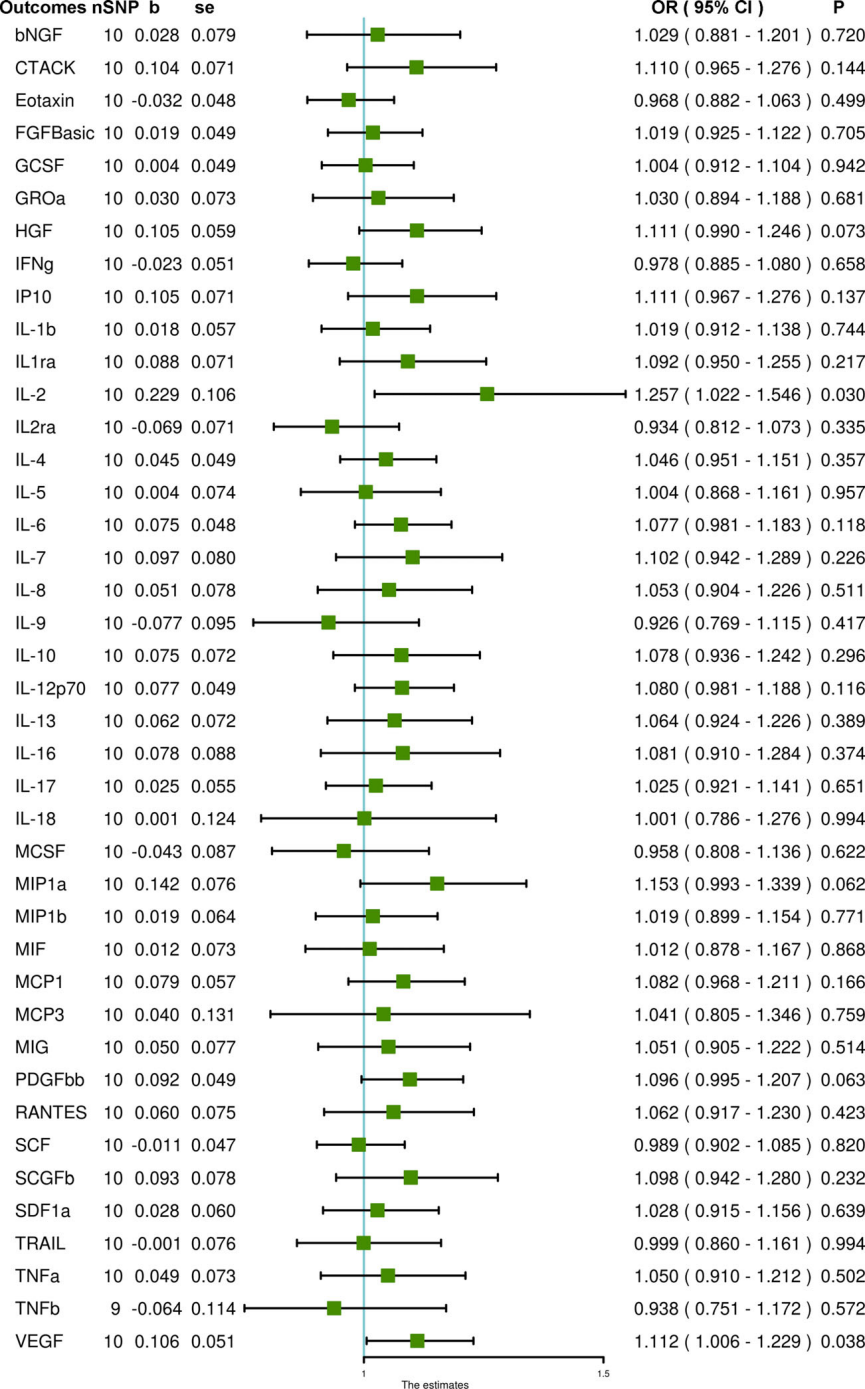
The association of PCOS with systemic inflammatory regulators using Mendelian randomization with SNPs reaching P < 5 × 10^–8^: the inverse-variance weighted (IVW) method was applied as the primary method for MR analysis. SNP, single nucleotide polymorphism; b, beta coefficient; se, standard error; OR, odds ratio; CI, confidence interval; MR-PRESSO, Mendelian randomization pleiotropy residual sum and outlier; bNGF, beta-nerve growth factor; CTACK, cutaneous T-cell attracting chemokine; FGFBasic, fibroblast growth factor basic; GCSF, granulocyte-colony stimulating factor; GROa, growth-regulated protein alpha; HGF, hepatocyte growth factor; IFNg, interferon gamma; IP10, interferon gamma-induced protein 10; IL-1b, interleukin-1-beta; IL1ra, interleukin-1-receptor antagonist; IL-2, interleukin-2; IL2ra, interleukin-2 receptor antagonist; IL-4, interleukin-4; IL-5, interleukin-5; IL-6, interleukin-6; IL-7, interleukin-7; IL-8, interleukin-8; IL-9, interleukin-9; IL-10, interleukin-10; IL-12p70, interleukin-12p70; IL-13, interleukin-13; IL-16, interleukin-16; IL-17, interleukin-17; IL-18, interleukin-18; MCSF, macrophage colony stimulating factor; MIP1a, macrophage inflammatory protein 1a; MIP1b, macrophage inflammatory protein 1b; MIF, macrophage migration inhibitory factor; MCP1, monocyte chemoattractant protein-1; MCP3, monocyte chemoattractant protein-3; MIG, monokine induced by gamma interferon; PDGFbb, platelet-derived growth factor BB; RANTES, regulated on activation, normal T cell expressed and secreted; SCF, stem cell factor; SCGFb, stem cell growth factor beta; SDF1a, stromal-cell-derived factor 1 alpha; TRAIL, TNF-related apoptosis-inducing ligand; TNFa, tumor necrosis factor alpha; TNFb, tumor necrosis factor beta; VEGF, vascular endothelial growth factor.

Using ten SNPs as IVs for PCOS, we demonstrated that genetically predicted PCOS is positively associated with IL-2 and VEGF levels through IVW (OR = 1.257, 95% CI = 1.022 – 1.546, P = 0.030 and OR = 1.112, 95% CI = 1.006 – 1.229, P = 0.038, respectively). Heterogeneity was not detected for VEGF (Cochran P value = 0.437) but was found for IL-2 (Cochran P value = 0.026). MR-Egger and MR-PRESSO did not detect potential horizontal pleiotropy for VEGF (P = 0.865 and P = 0.481, respectively). For IL-2, MR-Egger did not detect potential horizontal pleiotropy (P = 0.189), but MR-PRESSO detected pleiotropy (P = 0.033). After removing one outlier SNP (rs11225154), PCOS remained associated with increased IL-2 (OR = 1.199, 95% CI = 1.005 – 1.431, P = 0.044), and heterogeneity was also eliminated (Cochran P value = 0.169). In addition, PCOS was not associated with any other systemic inflammatory regulators in any analyses (**Figure 3** and **Supplementary Table S6**).

### Differences of Associated Systemic Inflammatory Regulators Levels Between PCOS Group and Control Group

A total of 30 patients were enrolled in this study, 15 in the PCOS group and 15 in the control group. Our findings suggested that PCOS patients had higher level of IL-2, SDF1-a, and VEGF, and lower level of IL-4, IL-17, and SCFGb, but the differences were not statically significant. And in the logistic regression analysis, IL-4 and IL-17 were associated with reduced risk of PCOS but none of the association was statically significant (**Supplementary Table S7**).

## Discussion

In this study, we performed a two-sample MR to determine the association of systemic inflammatory regulators with PCOS using the largest GWAS dataset to date. Our findings suggest that higher levels of IL-17 and SDF1a, as well as lower levels of SCGFb and IL-4, are associated with an increased risk of PCOS. Also, genetically predicted PCOS is related to increased levels of IL-2 and VEGF. To the best of our knowledge, this study is the first MR study that comprehensively evaluates the causal relationship between genetically predicted systemic inflammatory regulators and PCOS, and vice versa. Additionally, the results of MR study were validated by the results of our observational study.

Remarkably, a higher circulating level of IL-17 was found to be related to an increased risk of PCOS (OR = 1.794, 95% CI = 1.150 – 2.801, P = 0.010). IL-17 is a member of the pro-inflammatory cytokines, which are attributed to many inflammation-related diseases (22). A previous study has found that IL-17A (the founding member of the IL-17 family) levels were significantly increased in women with PCOS compared to healthy controls (p < 0.05) and that PCOS can be predicted by IL-17A at a cut-off value of 8.37 pg/mL with 44% sensitivity and 83% specificity in receiver operating characteristics (ROC) curve analysis (23). Another Iranian study found that IL-17A might be related to patients with a predisposition for PCOS because one SNP (rs2275913) was significantly different between the PCOS and control group (24). Thus, our findings are in accordance with previous studies in that a higher level of IL-17 may be involved in the pathogenesis of PCOS. SDF1a was another cytokine associated with a higher risk of PCOS (OR = 1.563, 95% CI = 1.055 – 2.315, P = 0.026). SDF1a, also known as CXCL12, is a chemokine member of the intercrine family and plays an important role in inflammation, angiogenesis, hematopoiesis and embryogenesis due to the activation and/or migration of most leukocytes, endothelial cells, hematopoietic progenitors and stem cells (25). But there are hardly any studies investigating the role of SDF1a in PCOS. Circulating SCGFb was found to be negatively associated with PCOS risk (OR = 0.838, 95% CI = 0.712 – 0.986, P = 0.034). SCGFb is a hematopoietic growth factor that exerts its cellular activity during the early stages of hematopoiesis (26). Nonetheless, our literature review did not find any reports of observational studies on the association between SCGFb and PCOS. The possibility of using SCGFb as a potential biomarker for PCOS may require further study. Our clinical analysis revealed that PCOS patients had higher level of SDF1a and lower level of SCGFb than the control group but the difference was not statistically significant that probably due to the limited sample size. Therefore, further studies are needed to continue investigating the relationship between SDF1a and SCGFb and PCOS. Moreover, our results indicate that IL-4 is inversely associated with the risk for PCOS (OR = 0.637, 95% CI = 0.413 – 0.983, P = 0.042). IL-4, an important type-2 T helper (Th2) type cytokine, plays an essential role in inflammation, antibody production modulation and the development of effector T-cell responses (27). Several studies have explored the relationship between IL-4 and PCOS. One study found that levels of IL-4 in patients with PCOS were significantly lower than those in healthy women, irrespective of BMI (28). Another study investigated the profiles of the type-1 T helper (Th1) (IFNg, IL-2) and Th2 (IL-4, IL-10) cytokines of CD3+CD4+ T lymphocyte subsets in follicular fluid (FF) and discovered that serum levels of IL-4 in patients with PCOS were reduced compared with those of controls (29). Further studies of these systemic inflammatory regulators and PCOS are warranted to explore the mechanisms underpinning these relationships.

Intriguingly, using bidirectional MR analysis, we also found that PCOS may be related to changes in the circulating levels of some cytokines. In our study, PCOS is associated with higher IL-2 and VEGF levels (OR = 1.257, 95% CI = 1.022 – 1.546, P = 0.030 and OR = 1.112, 95% CI = 1.006 – 1.229, P = 0.038, respectively). Although horizontal pleiotropy and heterogeneity were detected, they were diminished after removing an outlier SNP (rs11225154). Additionally, PCOS remained associated with increased IL-2 (OR = 1.199, 95% CI = 1.005 – 1.431, P = 0.044). IL-2, a Th1-type cytokine, participates in maintaining immune homeostasis through regulating regulatory T (Treg) cells, as well as the optimizing and adjusting of effector lymphocyte responses (30). Researchers have investigated the association between PCOS and IL-2 and found that obese PCOS patients have significantly increased levels of IL-2 in both serum and FF compared to healthy controls (29, 31). They have also found that PCOS patients exhibit decreased levels of Treg cells due to an inherent hypo-responsiveness to IL-2 (32). VEGF, known as endothelial cell mitogen, is an angiogenic growth factor that also leads to increased vascular permeability, expansion and sprouting (33). A previous systematic review indicated that circulating levels of VEGF were increased in PCOS patients, and the immunohistochemical (IHC) staining of VEGF in the ovarian theca and stroma of a polycystic ovary (PCO) were strong (34). Another review also concluded that PCOS patients had elevated levels of VEGF along with mild, chronic inflammation (35). Together with the results of our clinical analysis, we provide evidence for the increased levels of circulating IL-2 and VEGF in women with PCOS, but further studies are needed to fully understand the underlying molecular pathways.

The main strength of our study is that we adopted the MR method to investigate the association between systematic inflammatory regulators and PCOS. Therefore, our findings are less likely to be affected by inverse causality and potential confounding factors. Also, we extracted the SNPs of systemic inflammatory regulators and PCOS using the largest GWAS dataset to date. Additionally, the GWAS data on systematic inflammatory regulators were adjusted for body mass index, thus the bias was minimized. And we also validated the MR results by a clinical cohort. Our study also has, however, several limitations. First, we used a cut-off at a significance of P < 5 × 10^–6^ to extract SNPs from the GWAS data on systematic inflammatory regulators because only 17 had at least one genome-wide significant SNP when using P < 5 × 10^–8^ as the cut-off value; all 41 had at least one SNP when using the higher cut-off. Also, the finding for TNFb was based on a single SNP, and those of several cytokines, such as IL-1b, IL1ra, IL-5, IL-6, IL-10, IL-17, MCP3, MCSF, MIF and TNFa were based on two SNPs, which might have resulted in lower precision. Additionally, as the GWAS data on systemic inflammatory regulators did not provide the effect allele frequency (EAF), we were unable to identify palindromic SNPs to confirm whether the SNPs were aligned in the same direction for exposure and outcome. We did, however, apply MR-Egger and MR-PRESSO analyses to test for horizontal pleiotropy. Another problem is that the present MR study was entirely based on European ancestry, thus it is unclear whether our findings are still applicable to other races or regions. Moreover, since the relevant data of each PCOS phenotype was not available, we only explored the associations between systemic inflammatory regulators and the risk of PCOS, and we failed to stratify our results according to the different phenotypes of PCOS. Also we were not able to detect the significant difference of the expression of associated systematic inflammatory regulator between PCOS group and control group due to the limited sample size of our clinical study. Further studies focused on the association of cytokines and growth factors with PCOS are still needed.

In conclusion, using a two-sample MR approach, we provided evidence that higher genetically predicted circulating levels of IL-17 and SDF1a, as well as lower levels of SCGFb and IL-4, are associated with an increased risk of PCOS. Moreover, genetically predicted PCOS was found to be causally associated with increased levels of IL-2 and VEGF. Our results demonstrate the crucial role of cytokines in the pathogenesis of PCOS. Further studies are warranted to evaluate the possibility of these biomarkers as targets for PCOS prevention and treatment.

## Data Availability Statement

Publicly available datasets were analyzed in this study. This data can be found here: https://www.ebi.ac.uk/gwas/publications/27989323.

## Ethics Statement

The studies involving human participants were reviewed and approved by Ethical Review Board of West China Second University Hospital, Sichuan University. Written informed consent for participation was not required for this study in accordance with the national legislation and the institutional requirements.

## Author Contributions

Conceptualization: LQ, YW, HC and YZ. Data curation and formal analysis: RG and KC. Funding acquisition: LQ. Software and visualization: HC, YZ and YT. Supervision: SL, WX and YY. Writing of the original draft: HC and YZ. Review and editing: LQ and YW. HC and YZ verified the underlying data. All authors contributed to the article and approved the submitted version.

## Funding

This work was supported by the Sichuan Science and Technology Program [2020YFS0127] and was a Research Project of the Science & Technology Department of Sichuan Province (YZ, Grant No. 2021YJ0416). This work was also supported by the National Natural Science Foundation of China (YZ, Grant No. 82001496) and the China Postdoctoral Science Foundation (YZ, Grant No. 2020M680149, 2020T130087ZX).

## Conflict of Interest

The authors declare that the research was conducted in the absence of any commercial or financial relationships that could be construed as a potential conflict of interest.

## Publisher’s Note

All claims expressed in this article are solely those of the authors and do not necessarily represent those of their affiliated organizations, or those of the publisher, the editors and the reviewers. Any product that may be evaluated in this article, or claim that may be made by its manufacturer, is not guaranteed or endorsed by the publisher.
