# Supplementary material for: The Association Between Genetically Predicted Systemic Inflammatory Regulators and Polycystic Ovary Syndrome: A Mendelian Randomization Study
Source: Front Endocrinol (Lausanne). 2021 Sep 27;12:731569. doi: 10.3389/fendo.2021.731569 (PMC8503255; doi:10.3389/fendo.2021.731569)
Supplement: Supplementary file 1 [file DataSheet_1.zip › Data Sheet 1/supplementary materials/06_data(supplementary tableS2).docx]

**Supplementary Table S2. Characteristics of the genetic instrument variables for the systemic inflammatory regulators in the Mendelian randomization study at P < 5 × 10^–6^ significance level.**

| **Systematic inflammatory regulators** | **Number of SNPs** | **SNP** | **chr** | **pos** | **Effect allele** | **Other allele** | **Beta of exposure** | **SE of exposure** | **P of exposure** | **Beta of outcome** | **SE of outcome** | **P of outcome** | **F statistics** |
| --- | --- | --- | --- | --- | --- | --- | --- | --- | --- | --- | --- | --- | --- |
| bNGF | 5 | rs71641308 | 1 | 78086718 | C | T | -0.2043 | 0.0432 | 2.30E-06 | 0.11 | 0.069 | 0.11 | 22.36501736 |
|  |  | rs72780728 | 10 | 17603701 | G | A | -0.1883 | 0.0403 | 2.99E-06 | -0.044 | 0.053 | 0.41 | 21.83185045 |
|  |  | rs73472576 | 18 | 72124182 | C | T | 0.1181 | 0.0252 | 2.69E-06 | -0.031 | 0.035 | 0.38 | 21.96335664 |
|  |  | rs74966328 | 1 | 235707209 | G | A | 0.293 | 0.0618 | 2.13E-06 | -0.2 | 0.086 | 0.02 | 22.47803228 |
|  |  | rs7970581 | 12 | 113265248 | G | T | -0.138 | 0.0282 | 9.27E-07 | -0.026 | 0.039 | 0.51 | 23.94748755 |
| CTACK | 8 | rs12438356 | 15 | 69579577 | C | T | -0.1548 | 0.0337 | 4.46E-06 | -0.039 | 0.048 | 0.42 | 21.09998327 |
|  |  | rs141420735 | 9 | 34515043 | C | T | 0.3966 | 0.0789 | 5.82E-07 | -0.25 | 0.16 | 0.13 | 25.26686811 |
|  |  | rs2070074 | 9 | 34649442 | G | A | -0.4467 | 0.0374 | 1.79E-32 | -0.037 | 0.054 | 0.48 | 142.6555592 |
|  |  | rs2731671 | 5 | 176843441 | C | A | -0.1566 | 0.0338 | 3.45E-06 | 0.048 | 0.05 | 0.33 | 21.46595007 |
|  |  | rs55764737 | 15 | 61323414 | C | T | -0.5313 | 0.0972 | 4.62E-08 | -0.046 | 0.085 | 0.58 | 29.87769585 |
|  |  | rs57338032 | 15 | 78798939 | G | A | -0.1583 | 0.0317 | 6.23E-07 | -0.047 | 0.042 | 0.26 | 24.93694832 |
|  |  | rs76378954 | 9 | 33414861 | G | A | -0.4 | 0.0836 | 2.43E-06 | -0.23 | 0.11 | 0.036 | 22.89324878 |
|  |  | rs867811 | 9 | 34663798 | C | T | -0.1908 | 0.026 | 2.37E-13 | 0.032 | 0.032 | 0.32 | 53.85301775 |
| Eotaxin | 15 | rs11087905 | 21 | 25505329 | C | A | -0.0941 | 0.0189 | 5.48E-07 | 0.073 | 0.048 | 0.13 | 24.7888077 |
|  |  | rs116104576 | 3 | 43098795 | C | T | -0.3453 | 0.0428 | 5.71E-16 | -0.19 | 0.097 | 0.046 | 65.08870316 |
|  |  | rs1476670 | 1 | 44508195 | C | A | 0.1007 | 0.0217 | 3.51E-06 | 0.025 | 0.037 | 0.5 | 21.5347321 |
|  |  | rs2024050 | 7 | 75460393 | G | A | -0.1728 | 0.0303 | 1.10E-08 | -0.094 | 0.052 | 0.072 | 32.52387021 |
|  |  | rs2419841 | 10 | 115335983 | C | T | 0.1277 | 0.0279 | 4.98E-06 | 0.039 | 0.052 | 0.45 | 20.94948677 |
|  |  | rs2455949 | 21 | 25417866 | G | A | 0.0861 | 0.0177 | 1.30E-06 | -0.026 | 0.035 | 0.45 | 23.66245332 |
|  |  | rs342511 | 3 | 42578509 | G | A | -0.0927 | 0.0157 | 3.60E-09 | -0.042 | 0.031 | 0.18 | 34.86263134 |
|  |  | rs3823884 | 7 | 75544455 | C | A | -0.0909 | 0.0182 | 5.32E-07 | 0.025 | 0.035 | 0.47 | 24.94508513 |
|  |  | rs3960554 | 7 | 75845597 | G | T | -0.0798 | 0.0169 | 2.23E-06 | -0.1 | 0.043 | 0.021 | 22.29627814 |
|  |  | rs5754733 | 22 | 34269594 | C | A | 0.1042 | 0.0214 | 1.06E-06 | -0.041 | 0.037 | 0.26 | 23.70870818 |
|  |  | rs73070692 | 3 | 43035282 | G | A | -0.2826 | 0.0475 | 4.02E-09 | 0.062 | 0.1 | 0.55 | 35.39623712 |
|  |  | rs7433284 | 3 | 42872590 | G | A | 0.161 | 0.0165 | 1.61E-22 | -0.096 | 0.032 | 0.0028 | 95.21028466 |
|  |  | rs75426604 | 14 | 35857714 | C | A | 0.1366 | 0.0291 | 2.53E-06 | 0.03 | 0.049 | 0.54 | 22.03512004 |
|  |  | rs79722574 | 17 | 32619052 | C | T | 0.1113 | 0.0228 | 1.06E-06 | 0.081 | 0.042 | 0.054 | 23.82981302 |
|  |  | rs80341932 | 18 | 56915928 | G | A | -0.1016 | 0.0205 | 6.69E-07 | 0.063 | 0.042 | 0.13 | 24.56290303 |
| FGFBasic | 3 | rs747334 | 10 | 92744744 | G | A | -0.0751 | 0.0164 | 4.53E-06 | -0.046 | 0.031 | 0.14 | 20.9696981 |
|  |  | rs75168112 | 18 | 71086067 | C | T | 0.1001 | 0.0214 | 3.00E-06 | 0.03 | 0.048 | 0.53 | 21.87966198 |
|  |  | rs9903590 | 17 | 34238290 | C | T | -0.1313 | 0.0268 | 8.01E-07 | 0.048 | 0.052 | 0.36 | 24.00268712 |
| GCSF | 3 | rs11903143 | 2 | 29592460 | G | A | -0.087 | 0.0176 | 6.35E-07 | -0.029 | 0.035 | 0.41 | 24.43504649 |
|  |  | rs1817411 | 8 | 98598328 | C | T | -0.089 | 0.0191 | 3.10E-06 | 0.039 | 0.037 | 0.29 | 21.71267235 |
|  |  | rs76287671 | 19 | 44122296 | C | T | -0.0938 | 0.0189 | 6.92E-07 | 0.077 | 0.043 | 0.073 | 24.63100137 |
| GROa | 6 | rs118158560 | 7 | 38146610 | G | A | -0.2703 | 0.0594 | 3.42E-06 | 0.048 | 0.079 | 0.54 | 20.70709621 |
|  |  | rs12727188 | 1 | 159486328 | G | T | 0.2159 | 0.0359 | 2.23E-09 | -0.035 | 0.052 | 0.51 | 36.1673249 |
|  |  | rs150194856 | 1 | 159191342 | C | T | 0.4375 | 0.0923 | 2.19E-06 | -0.069 | 0.088 | 0.43 | 22.46739458 |
|  |  | rs2422841 | 20 | 3080352 | G | A | 0.1657 | 0.0361 | 4.66E-06 | -0.13 | 0.06 | 0.033 | 21.06835429 |
|  |  | rs4483403 | 1 | 159543683 | G | T | -0.1534 | 0.033 | 1.82E-06 | 0.058 | 0.054 | 0.28 | 21.60841139 |
|  |  | rs73020724 | 1 | 159581435 | C | A | -0.2617 | 0.0544 | 2.45E-06 | -0.09 | 0.11 | 0.4 | 23.14246661 |
| HGF | 8 | rs10007119 | 4 | 3473066 | G | A | 0.0851 | 0.0164 | 2.02E-07 | 0.018 | 0.032 | 0.57 | 26.92597412 |
|  |  | rs11060254 | 12 | 129815569 | G | A | 0.08 | 0.0167 | 1.58E-06 | -0.017 | 0.034 | 0.61 | 22.94811574 |
|  |  | rs150322232 | 7 | 7930374 | G | A | -0.2104 | 0.0463 | 4.89E-06 | -0.06 | 0.12 | 0.61 | 20.65044853 |
|  |  | rs1698249 | 14 | 84356186 | C | A | 0.1698 | 0.0372 | 4.09E-06 | 0.053 | 0.053 | 0.32 | 20.83480749 |
|  |  | rs2003620 | 7 | 134479484 | C | T | -0.2279 | 0.0489 | 2.83E-06 | 0.039 | 0.07 | 0.58 | 21.7205557 |
|  |  | rs3748034 | 4 | 3446091 | G | T | -0.1495 | 0.0234 | 1.81E-10 | 0.046 | 0.045 | 0.31 | 40.81790123 |
|  |  | rs5745687 | 7 | 81359051 | C | T | 0.3072 | 0.0406 | 2.75E-14 | 0.13 | 0.064 | 0.045 | 57.25195952 |
|  |  | rs62481625 | 7 | 155987460 | C | T | -0.1091 | 0.0225 | 1.18E-06 | 0.079 | 0.039 | 0.042 | 23.51172346 |
| IFNg | 4 | rs10487554 | 7 | 149367686 | G | A | 0.0895 | 0.0183 | 1.09E-06 | 0.032 | 0.035 | 0.36 | 23.91904805 |
|  |  | rs112783231 | 8 | 68123274 | G | A | 0.2408 | 0.0511 | 1.96E-06 | 0.24 | 0.13 | 0.075 | 22.20604241 |
|  |  | rs11843756 | 13 | 49254892 | G | T | -0.184 | 0.0393 | 3.09E-06 | -0.078 | 0.087 | 0.37 | 21.9205045 |
|  |  | rs12420286 | 11 | 103777894 | C | T | -0.2376 | 0.0501 | 2.08E-06 | 0.093 | 0.078 | 0.23 | 22.49144824 |
| IL-1b | 2 | rs4786740 | 16 | 5675198 | C | A | -0.0845 | 0.0202 | 4.67E-06 | 0.029 | 0.034 | 0.39 | 17.49889717 |
|  |  | rs62015704 | 16 | 7467907 | G | A | -0.1082 | 0.0283 | 2.09E-06 | 0.068 | 0.05 | 0.18 | 14.61778771 |
| IL1ra | 2 | rs56134659 | 3 | 129058985 | G | A | 0.1117 | 0.0237 | 2.44E-06 | -0.046 | 0.034 | 0.18 | 22.21312468 |
|  |  | rs9623661 | 22 | 43093376 | C | T | 0.1966 | 0.0426 | 3.86E-06 | -0.034 | 0.06 | 0.57 | 21.29844167 |
| IL-2 | 7 | rs12051139 | 16 | 86918674 | C | T | 0.1131 | 0.0247 | 4.76E-06 | -0.065 | 0.032 | 0.045 | 20.966759 |
|  |  | rs170117 | 4 | 55390380 | C | T | 0.1617 | 0.0349 | 3.87E-06 | -0.058 | 0.049 | 0.23 | 21.46689272 |
|  |  | rs2807544 | 1 | 15204245 | G | A | -0.1175 | 0.0253 | 3.41E-06 | -0.032 | 0.033 | 0.34 | 21.56923245 |
|  |  | rs4634519 | 7 | 67192928 | G | A | 0.1261 | 0.0269 | 2.77E-06 | 0.04 | 0.034 | 0.24 | 21.97483451 |
|  |  | rs62124990 | 2 | 19238636 | G | T | 0.6961 | 0.1495 | 3.22E-06 | -0.17 | 0.15 | 0.27 | 21.68008009 |
|  |  | rs7615304 | 3 | 156675703 | G | A | 0.1172 | 0.0242 | 1.21E-06 | -0.046 | 0.032 | 0.15 | 23.45440885 |
|  |  | rs80336398 | 3 | 64060934 | C | T | -0.4001 | 0.0858 | 2.82E-06 | 0.12 | 0.12 | 0.33 | 21.74515597 |
| IL2ra | 6 | rs115360066 | 5 | 9823768 | G | A | -0.1867 | 0.0379 | 8.06E-07 | -0.08 | 0.054 | 0.14 | 24.26667177 |
|  |  | rs2182409 | 10 | 6209218 | C | T | -0.12 | 0.0251 | 1.66E-06 | 0.084 | 0.036 | 0.02 | 22.85678005 |
|  |  | rs34507893 | 10 | 6124598 | G | A | -0.7344 | 0.1164 | 2.91E-10 | -0.44 | 0.26 | 0.088 | 39.8069933 |
|  |  | rs4733117 | 8 | 32137610 | C | A | -0.1369 | 0.0292 | 2.63E-06 | 0.048 | 0.044 | 0.28 | 21.98068352 |
|  |  | rs61705228 | 4 | 101196302 | C | T | -0.3303 | 0.0716 | 3.99E-06 | 0.13 | 0.081 | 0.11 | 21.28095448 |
|  |  | rs7077067 | 10 | 6132692 | C | T | 0.1101 | 0.0241 | 5.00E-06 | -0.025 | 0.032 | 0.44 | 20.87086999 |
| IL-4 | 3 | rs17713451 | 7 | 151162472 | G | A | -0.1274 | 0.0253 | 4.97E-07 | 0.041 | 0.045 | 0.37 | 25.35699667 |
|  |  | rs7613691 | 3 | 147653591 | G | A | -0.1775 | 0.0384 | 4.05E-06 | 0.058 | 0.069 | 0.4 | 21.36654324 |
|  |  | rs9508291 | 13 | 29710620 | C | T | 0.1676 | 0.0359 | 3.03E-06 | -0.13 | 0.07 | 0.068 | 21.79511332 |
| IL-5 | 2 | rs6737109 | 2 | 23179531 | C | T | -0.116 | 0.0247 | 2.40E-06 | 0.041 | 0.032 | 0.2 | 22.05576226 |
|  |  | rs72831687 | 6 | 16092360 | G | A | 0.5239 | 0.1109 | 1.69E-06 | -0.31 | 0.2 | 0.12 | 22.31689163 |
| IL-6 | 2 | rs72648871 | 17 | 45816108 | C | T | -0.1207 | 0.0253 | 1.74E-06 | -0.043 | 0.062 | 0.49 | 22.76006499 |
|  |  | rs73273528 | 20 | 50431113 | C | T | -0.2672 | 0.0553 | 9.58E-07 | -0.1 | 0.082 | 0.22 | 23.34654637 |
| IL-7 | 8 | rs117509142 | 8 | 87134083 | C | T | 0.327 | 0.0688 | 1.99E-06 | 0.073 | 0.084 | 0.39 | 22.59012473 |
|  |  | rs141425475 | 5 | 17679165 | C | T | 0.4781 | 0.1016 | 2.53E-06 | 0.063 | 0.12 | 0.61 | 22.14369401 |
|  |  | rs17091524 | 14 | 56948759 | C | T | -0.4924 | 0.1013 | 1.91E-06 | 0.14 | 0.1 | 0.19 | 23.62746877 |
|  |  | rs28793375 | 8 | 41415618 | C | T | -0.1638 | 0.0361 | 4.46E-06 | -0.04 | 0.045 | 0.38 | 20.58796357 |
|  |  | rs62006410 | 14 | 103007935 | C | T | 0.1557 | 0.0303 | 3.39E-07 | -0.17 | 0.046 | 0.00022 | 26.40535242 |
|  |  | rs73610561 | 19 | 55060940 | C | T | -0.2874 | 0.0624 | 3.96E-06 | 0.053 | 0.074 | 0.47 | 21.21311021 |
|  |  | rs75904417 | 2 | 168653321 | C | A | 0.1698 | 0.0349 | 1.16E-06 | -0.075 | 0.049 | 0.12 | 23.67143127 |
|  |  | rs77981494 | 16 | 17544866 | C | T | 0.5178 | 0.1064 | 1.07E-06 | -0.12 | 0.12 | 0.31 | 23.68322474 |
| IL-8 | 3 | rs11634944 | 15 | 25183093 | C | T | 0.1214 | 0.0252 | 1.29E-06 | -0.048 | 0.033 | 0.14 | 23.20792391 |
|  |  | rs141926526 | 7 | 32848640 | C | A | 0.6149 | 0.1308 | 2.57E-06 | 0.053 | 0.082 | 0.51 | 22.10006231 |
|  |  | rs2673604 | 8 | 133411607 | C | A | 0.1266 | 0.0255 | 7.02E-07 | 0.044 | 0.035 | 0.21 | 24.6483045 |
| IL-9 | 3 | rs4880409 | 10 | 134330220 | C | T | 0.3355 | 0.0723 | 3.50E-06 | -0.071 | 0.073 | 0.33 | 21.53319406 |
|  |  | rs61867538 | 11 | 1524506 | C | T | -0.3566 | 0.0774 | 3.93E-06 | 0.08 | 0.13 | 0.55 | 21.22661565 |
|  |  | rs7242404 | 18 | 12741267 | G | A | 0.1228 | 0.0264 | 3.27E-06 | 0.04 | 0.035 | 0.25 | 21.6365932 |
| IL-10 | 2 | rs465757 | 20 | 15580283 | G | A | -0.084 | 0.0174 | 1.17E-06 | 0.03 | 0.035 | 0.38 | 23.30558859 |
|  |  | rs6085948 | 20 | 7233350 | G | A | -0.098 | 0.0202 | 1.25E-06 | 0.02 | 0.037 | 0.59 | 23.53690815 |
| IL-12p70 | 3 | rs12770839 | 10 | 65297720 | C | A | -0.0885 | 0.0172 | 2.92E-07 | 0.029 | 0.034 | 0.39 | 26.47461466 |
|  |  | rs41282644 | 6 | 43753722 | G | A | -0.1473 | 0.0304 | 1.05E-06 | -0.18 | 0.11 | 0.11 | 23.47785016 |
|  |  | rs782107 | 12 | 58833530 | G | A | -0.075 | 0.0156 | 1.60E-06 | 0.022 | 0.031 | 0.48 | 23.11390533 |
| IL-13 | 8 | rs117795020 | 9 | 90084152 | G | A | 0.3522 | 0.0716 | 9.86E-07 | 0.13 | 0.15 | 0.39 | 24.19650604 |
|  |  | rs12623722 | 2 | 23178683 | G | A | 0.1185 | 0.0258 | 4.19E-06 | -0.033 | 0.036 | 0.36 | 21.09586263 |
|  |  | rs138854806 | 6 | 43780360 | G | A | 0.4256 | 0.0845 | 6.47E-07 | 0.39 | 0.17 | 0.023 | 25.3682098 |
|  |  | rs139083458 | 5 | 26160518 | C | T | -0.9902 | 0.2107 | 2.81E-06 | -0.34 | 0.14 | 0.011 | 22.08598499 |
|  |  | rs2370048 | 3 | 132960738 | G | A | 0.1235 | 0.0266 | 3.35E-06 | -0.047 | 0.035 | 0.18 | 21.55612245 |
|  |  | rs27949 | 5 | 58550823 | C | T | 0.1168 | 0.0252 | 3.43E-06 | -0.044 | 0.033 | 0.18 | 21.48248929 |
|  |  | rs7073807 | 10 | 69153428 | C | T | -0.1682 | 0.0356 | 2.37E-06 | 0.073 | 0.052 | 0.16 | 22.32297058 |
|  |  | rs75995699 | 6 | 5140856 | G | A | -0.3319 | 0.0698 | 2.64E-06 | 0.09 | 0.095 | 0.34 | 22.61016125 |
| IL-16 | 7 | rs117916513 | 11 | 121264274 | G | A | 0.502 | 0.0986 | 3.79E-07 | 0.13 | 0.14 | 0.33 | 25.92111056 |
|  |  | rs12765671 | 10 | 106684169 | G | A | 0.6023 | 0.1318 | 4.84E-06 | -0.12 | 0.11 | 0.28 | 20.883097 |
|  |  | rs144691581 | 15 | 96953325 | G | A | -0.4882 | 0.0967 | 4.20E-07 | -0.11 | 0.18 | 0.55 | 25.48840164 |
|  |  | rs28515781 | 4 | 113683867 | C | T | -0.1332 | 0.0285 | 3.03E-06 | -0.042 | 0.038 | 0.28 | 21.8433241 |
|  |  | rs3848180 | 15 | 81596590 | G | T | -0.1223 | 0.0248 | 9.42E-07 | 0.065 | 0.031 | 0.038 | 24.31921501 |
|  |  | rs4253283 | 4 | 187165211 | C | T | -0.146 | 0.0262 | 1.75E-08 | -0.038 | 0.033 | 0.25 | 31.05296894 |
|  |  | rs4513633 | 4 | 113570639 | C | A | 0.2239 | 0.0453 | 7.44E-07 | 0.051 | 0.044 | 0.25 | 24.42934277 |
| IL-17 | 2 | rs184080173 | 12 | 77725204 | C | T | -0.2384 | 0.0471 | 4.19E-07 | -0.14 | 0.061 | 0.026 | 25.61950226 |
|  |  | rs187475560 | 4 | 161274563 | C | T | 0.2434 | 0.052 | 3.29E-06 | 0.14 | 0.12 | 0.25 | 21.90960059 |
| IL-18 | 10 | rs10414578 | 19 | 55146070 | C | T | 0.1771 | 0.035 | 4.16E-07 | -0.071 | 0.049 | 0.15 | 25.6036 |
|  |  | rs116656892 | 5 | 68186028 | C | T | -0.5298 | 0.0925 | 1.05E-08 | 0.13 | 0.1 | 0.22 | 32.80503024 |
|  |  | rs11700536 | 21 | 44558687 | C | T | -0.1156 | 0.025 | 4.21E-06 | -0.048 | 0.034 | 0.16 | 21.381376 |
|  |  | rs117266781 | 7 | 41301020 | C | T | -0.6841 | 0.1468 | 3.15E-06 | -0.23 | 0.19 | 0.22 | 21.71636186 |
|  |  | rs141091241 | 11 | 112111460 | C | T | 0.4122 | 0.0728 | 1.83E-08 | 0.13 | 0.096 | 0.17 | 32.05923952 |
|  |  | rs17229943 | 5 | 68682536 | C | A | 0.312 | 0.0463 | 1.62E-11 | 0.072 | 0.082 | 0.38 | 45.40955082 |
|  |  | rs1852105 | 7 | 63725595 | C | T | -0.3036 | 0.0661 | 4.32E-06 | -0.038 | 0.072 | 0.59 | 21.09602422 |
|  |  | rs1979967 | 15 | 79659613 | C | T | -0.1402 | 0.0286 | 9.45E-07 | 0.02 | 0.036 | 0.58 | 24.03056384 |
|  |  | rs2729385 | 11 | 57262993 | G | A | -0.1231 | 0.0262 | 3.79E-06 | 0.038 | 0.036 | 0.29 | 22.07565119 |
|  |  | rs78716465 | 20 | 40643726 | G | A | -0.3265 | 0.0682 | 1.63E-06 | 0.11 | 0.094 | 0.24 | 22.91910329 |
| IP10 | 7 | rs10809307 | 9 | 11045908 | C | T | -0.1305 | 0.0282 | 3.64E-06 | -0.062 | 0.033 | 0.059 | 21.41523314 |
|  |  | rs11626201 | 14 | 36980700 | C | A | -0.1162 | 0.0245 | 1.93E-06 | 0.053 | 0.033 | 0.11 | 22.49469388 |
|  |  | rs143799975 | 4 | 76807015 | G | A | 0.7984 | 0.1637 | 1.00E-06 | -0.31 | 0.15 | 0.042 | 23.78722047 |
|  |  | rs397816 | 22 | 22728326 | C | T | -0.1237 | 0.0249 | 7.90E-07 | -0.039 | 0.055 | 0.48 | 24.6797471 |
|  |  | rs7645625 | 3 | 146574037 | G | T | 0.1086 | 0.0237 | 4.41E-06 | 0.028 | 0.032 | 0.39 | 20.99727608 |
|  |  | rs7690978 | 4 | 75991844 | G | A | -0.1247 | 0.0259 | 1.46E-06 | 0.036 | 0.037 | 0.34 | 23.18106468 |
|  |  | rs9450351 | 6 | 86624320 | C | T | 0.2768 | 0.0489 | 1.48E-08 | 0.047 | 0.062 | 0.45 | 32.0416191 |
| MCP1 | 12 | rs10145849 | 14 | 82941991 | G | A | 0.0755 | 0.0162 | 3.41E-06 | 0.041 | 0.032 | 0.2 | 21.72020271 |
|  |  | rs10744620 | 12 | 3739094 | C | T | -0.0788 | 0.0161 | 9.91E-07 | -0.05 | 0.032 | 0.12 | 23.95524864 |
|  |  | rs116425179 | 3 | 45598703 | G | A | 0.1476 | 0.0255 | 5.68E-09 | -0.058 | 0.07 | 0.41 | 33.50366782 |
|  |  | rs141676607 | 3 | 46653244 | C | T | -0.3128 | 0.064 | 9.48E-07 | -0.17 | 0.12 | 0.14 | 23.88765625 |
|  |  | rs145155829 | 1 | 44165646 | C | T | 0.2153 | 0.0463 | 3.72E-06 | 0.14 | 0.1 | 0.16 | 21.62350433 |
|  |  | rs2281300 | 1 | 159156285 | C | T | 0.0889 | 0.017 | 1.71E-07 | -0.019 | 0.037 | 0.61 | 27.3467474 |
|  |  | rs34190208 | 3 | 46736217 | C | T | -0.1076 | 0.022 | 9.91E-07 | 0.02 | 0.036 | 0.58 | 23.92099174 |
|  |  | rs35333710 | 1 | 159172854 | G | A | -0.1476 | 0.0268 | 3.71E-08 | -0.074 | 0.055 | 0.18 | 30.33214524 |
|  |  | rs41338844 | 3 | 46272951 | G | A | -0.2195 | 0.0462 | 3.22E-06 | 0.067 | 0.11 | 0.53 | 22.57278256 |
|  |  | rs56212190 | 1 | 42168539 | C | T | -0.181 | 0.0373 | 9.85E-07 | -0.038 | 0.074 | 0.6 | 23.54721158 |
|  |  | rs7517040 | 1 | 158859133 | G | A | 0.0987 | 0.0191 | 2.44E-07 | -0.019 | 0.037 | 0.6 | 26.70346208 |
|  |  | rs952287 | 3 | 45899615 | G | A | 0.0904 | 0.0175 | 1.92E-07 | -0.034 | 0.034 | 0.32 | 26.68460408 |
| MCP3 | 2 | rs10892381 | 11 | 119400817 | C | T | -0.2412 | 0.0476 | 3.56E-07 | -0.058 | 0.035 | 0.1 | 25.67678836 |
|  |  | rs7008123 | 8 | 10434018 | C | T | 0.2065 | 0.0447 | 3.76E-06 | -0.041 | 0.035 | 0.25 | 21.34150614 |
| MCSF | 2 | rs117867915 | 18 | 42210043 | C | T | -0.5272 | 0.1098 | 1.61E-06 | -0.27 | 0.17 | 0.11 | 23.05399119 |
|  |  | rs9387100 | 6 | 113102954 | C | T | 0.1352 | 0.0292 | 4.07E-06 | 0.029 | 0.033 | 0.38 | 21.43816851 |
| MIF | 2 | rs12594190 | 15 | 25036455 | G | A | -0.1355 | 0.0267 | 3.70E-07 | -0.025 | 0.04 | 0.53 | 25.75467463 |
|  |  | rs13142904 | 4 | 54318414 | C | T | 0.223 | 0.0425 | 2.56E-07 | -0.043 | 0.07 | 0.53 | 27.5316263 |
| MIG | 10 | rs111607343 | 19 | 897855 | G | A | 0.521 | 0.1119 | 2.83E-06 | -0.21 | 0.12 | 0.081 | 21.67780341 |
|  |  | rs11177248 | 12 | 68875886 | G | A | -0.3073 | 0.067 | 4.45E-06 | -0.1 | 0.072 | 0.17 | 21.03659835 |
|  |  | rs112337562 | 14 | 93131570 | G | T | 0.37 | 0.0796 | 2.98E-06 | -0.13 | 0.16 | 0.42 | 21.60614631 |
|  |  | rs112861654 | 21 | 43599172 | G | A | 0.2765 | 0.0529 | 1.81E-07 | 0.039 | 0.075 | 0.6 | 27.31988879 |
|  |  | rs1796086 | 7 | 70648715 | C | T | 0.2096 | 0.0403 | 2.23E-07 | 0.12 | 0.054 | 0.033 | 27.05032357 |
|  |  | rs41272086 | 6 | 161008646 | G | A | 0.2226 | 0.0415 | 7.43E-08 | 0.082 | 0.054 | 0.13 | 28.77094498 |
|  |  | rs5752128 | 22 | 25718623 | C | T | 0.1685 | 0.0369 | 4.34E-06 | -0.069 | 0.066 | 0.3 | 20.85196936 |
|  |  | rs62562991 | 9 | 98736059 | G | A | -0.6236 | 0.126 | 8.40E-07 | -0.15 | 0.1 | 0.16 | 24.49464349 |
|  |  | rs77086208 | 14 | 70619491 | C | T | -0.3226 | 0.0698 | 3.83E-06 | 0.17 | 0.18 | 0.33 | 21.36081806 |
|  |  | rs816960 | 13 | 108522521 | C | T | 0.1224 | 0.0244 | 5.01E-07 | 0.086 | 0.036 | 0.019 | 25.16420317 |
| MIP1a | 5 | rs10835056 | 11 | 26697017 | G | T | -0.1194 | 0.0254 | 2.60E-06 | 0.077 | 0.035 | 0.026 | 22.09740219 |
|  |  | rs12690897 | 7 | 85346177 | G | A | -0.1248 | 0.0262 | 2.11E-06 | -0.047 | 0.035 | 0.17 | 22.68958685 |
|  |  | rs57786342 | 14 | 69260028 | G | A | -0.1314 | 0.0285 | 4.06E-06 | 0.084 | 0.04 | 0.034 | 21.25695291 |
|  |  | rs60198979 | 22 | 43646704 | G | A | 0.2146 | 0.0458 | 2.62E-06 | -0.038 | 0.058 | 0.52 | 21.95474915 |
|  |  | rs6900267 | 6 | 380341 | C | A | 0.2429 | 0.0519 | 2.89E-06 | 0.098 | 0.068 | 0.15 | 21.9038428 |
| MIP1b | 67 | rs11080371 | 17 | 34375136 | C | T | -0.1035 | 0.0164 | 3.20E-10 | 0.03 | 0.034 | 0.38 | 39.82841315 |
|  |  | rs11130043 | 3 | 45111239 | G | A | 0.0731 | 0.0157 | 3.22E-06 | 0.017 | 0.032 | 0.6 | 21.6788105 |
|  |  | rs111721971 | 17 | 35073020 | G | T | 0.219 | 0.0471 | 3.18E-06 | 0.088 | 0.084 | 0.29 | 21.61953832 |
|  |  | rs112078619 | 17 | 33836531 | G | A | -0.2675 | 0.0486 | 5.46E-08 | -0.15 | 0.11 | 0.2 | 30.29528442 |
|  |  | rs112311659 | 3 | 46799444 | C | A | -0.2744 | 0.0588 | 3.03E-06 | -0.079 | 0.13 | 0.53 | 21.77777778 |
|  |  | rs113010081 | 3 | 46457412 | C | T | 0.5954 | 0.0236 | 3.85E-140 | 0.12 | 0.051 | 0.014 | 636.4930336 |
|  |  | rs113699401 | 17 | 33668796 | G | A | -0.1474 | 0.0212 | 3.35E-12 | -0.033 | 0.049 | 0.5 | 48.34184763 |
|  |  | rs114164513 | 3 | 45272343 | C | T | 0.3269 | 0.0351 | 2.09E-20 | -0.09 | 0.092 | 0.33 | 86.73923913 |
|  |  | rs116237296 | 1 | 87045516 | G | A | -0.5437 | 0.1115 | 7.23E-07 | 0.24 | 0.2 | 0.24 | 23.77765006 |
|  |  | rs11651172 | 17 | 34270288 | G | A | 0.1366 | 0.0245 | 2.11E-08 | -0.1 | 0.051 | 0.041 | 31.08631404 |
|  |  | rs117023032 | 17 | 32887783 | G | A | 0.2448 | 0.052 | 2.11E-06 | 0.28 | 0.21 | 0.18 | 22.16236686 |
|  |  | rs117028362 | 17 | 35342587 | G | A | 0.3286 | 0.071 | 4.50E-06 | 0.27 | 0.19 | 0.14 | 21.41994842 |
|  |  | rs117120228 | 17 | 33780205 | G | A | 0.3405 | 0.0743 | 4.27E-06 | -0.26 | 0.23 | 0.26 | 21.00180419 |
|  |  | rs117394484 | 17 | 34363701 | C | T | 0.421 | 0.0783 | 7.69E-08 | 0.086 | 0.16 | 0.6 | 28.9095058 |
|  |  | rs117620244 | 17 | 33648381 | C | T | 0.3528 | 0.0495 | 1.87E-12 | -0.1 | 0.16 | 0.52 | 50.79801653 |
|  |  | rs1252860 | 17 | 35125060 | G | A | -0.1232 | 0.0176 | 2.60E-12 | 0.024 | 0.035 | 0.5 | 49 |
|  |  | rs12601380 | 17 | 34904985 | C | A | 0.2159 | 0.0163 | 4.21E-40 | -0.018 | 0.032 | 0.57 | 175.4405887 |
|  |  | rs1437220 | 17 | 32791974 | C | T | -0.1478 | 0.0315 | 3.53E-06 | -0.062 | 0.094 | 0.51 | 22.01545981 |
|  |  | rs145032274 | 3 | 45610259 | C | T | -0.0796 | 0.0171 | 4.32E-06 | -0.094 | 0.04 | 0.018 | 21.66875278 |
|  |  | rs146394760 | 17 | 35247215 | C | T | 0.1517 | 0.0307 | 5.71E-07 | -0.094 | 0.091 | 0.3 | 24.41711848 |
|  |  | rs148561432 | 17 | 33831939 | G | A | 0.2703 | 0.0409 | 6.78E-11 | 0.074 | 0.097 | 0.45 | 43.67626329 |
|  |  | rs148883658 | 17 | 34992469 | C | A | 0.5294 | 0.046 | 1.62E-30 | -0.17 | 0.25 | 0.51 | 132.4500756 |
|  |  | rs150641077 | 17 | 34195451 | G | A | 0.2991 | 0.0492 | 1.25E-09 | 0.28 | 0.17 | 0.096 | 36.95750297 |
|  |  | rs1564708 | 17 | 34825482 | C | T | 0.1744 | 0.0188 | 2.87E-20 | -0.059 | 0.047 | 0.21 | 86.05522861 |
|  |  | rs159309 | 17 | 32712231 | C | T | -0.1283 | 0.0259 | 6.71E-07 | -0.055 | 0.045 | 0.23 | 24.5388262 |
|  |  | rs17138331 | 7 | 7866368 | G | A | 0.1391 | 0.0295 | 2.26E-06 | -0.042 | 0.052 | 0.41 | 22.23362252 |
|  |  | rs17693183 | 17 | 34964290 | G | A | 0.5795 | 0.0795 | 8.93E-13 | -0.19 | 0.2 | 0.35 | 53.13401369 |
|  |  | rs192429455 | 3 | 43442879 | G | A | 0.2082 | 0.0423 | 8.53E-07 | -0.34 | 0.17 | 0.046 | 24.22594437 |
|  |  | rs1979671 | 3 | 46274215 | C | T | -0.1216 | 0.017 | 7.86E-13 | -0.053 | 0.033 | 0.11 | 51.16456747 |
|  |  | rs1994089 | 17 | 33516594 | C | T | 0.1116 | 0.0162 | 5.50E-12 | -0.036 | 0.032 | 0.26 | 47.45679012 |
|  |  | rs2003485 | 3 | 50176589 | C | T | -0.0873 | 0.017 | 2.72E-07 | -0.023 | 0.034 | 0.49 | 26.37124567 |
|  |  | rs2239692 | 3 | 46488854 | C | T | -0.1283 | 0.0203 | 2.39E-10 | 0.036 | 0.049 | 0.46 | 39.94489068 |
|  |  | rs2411190 | 17 | 34992830 | G | A | -0.1822 | 0.0207 | 2.78E-18 | -0.055 | 0.042 | 0.19 | 77.4740134 |
|  |  | rs2531742 | 3 | 45841068 | G | A | -0.1922 | 0.0161 | 1.02E-32 | 0.021 | 0.033 | 0.51 | 142.5131746 |
|  |  | rs2671831 | 17 | 33727229 | G | A | -0.0715 | 0.0155 | 4.50E-06 | -0.054 | 0.031 | 0.083 | 21.27887617 |
|  |  | rs2673050 | 3 | 45739807 | G | T | -0.1314 | 0.0161 | 3.14E-16 | -0.018 | 0.031 | 0.57 | 66.60993017 |
|  |  | rs281749 | 8 | 108638645 | C | T | -0.0799 | 0.0171 | 3.17E-06 | -0.043 | 0.033 | 0.19 | 21.83239287 |
|  |  | rs35715602 | 17 | 35023713 | C | T | -0.1809 | 0.0342 | 1.47E-07 | 0.091 | 0.092 | 0.32 | 27.97853186 |
|  |  | rs35933743 | 17 | 32970484 | G | T | 0.126 | 0.0239 | 1.50E-07 | 0.074 | 0.051 | 0.14 | 27.79363106 |
|  |  | rs4682782 | 3 | 45394940 | G | A | -0.0759 | 0.016 | 2.32E-06 | -0.034 | 0.032 | 0.29 | 22.50316406 |
|  |  | rs4795162 | 17 | 35236530 | G | A | -0.1261 | 0.0158 | 1.14E-15 | 0.046 | 0.031 | 0.14 | 63.69656305 |
|  |  | rs4796235 | 17 | 35014357 | G | A | -0.0919 | 0.0179 | 2.81E-07 | 0.034 | 0.043 | 0.42 | 26.35875909 |
|  |  | rs55771110 | 3 | 47346717 | G | A | -0.1114 | 0.019 | 4.40E-09 | 0.06 | 0.045 | 0.18 | 34.3766205 |
|  |  | rs60516659 | 17 | 34403297 | G | A | -0.2691 | 0.0248 | 3.64E-27 | 0.063 | 0.053 | 0.23 | 117.7400007 |
|  |  | rs62243190 | 3 | 45524938 | C | T | -0.4572 | 0.0409 | 4.17E-29 | 0.45 | 0.31 | 0.15 | 124.9585069 |
|  |  | rs6505501 | 17 | 34347238 | C | T | 0.1556 | 0.0191 | 3.71E-16 | 0.047 | 0.043 | 0.27 | 66.36704038 |
|  |  | rs6802820 | 3 | 46630759 | C | T | 0.1233 | 0.0263 | 2.88E-06 | -0.073 | 0.057 | 0.2 | 21.97934046 |
|  |  | rs7215719 | 17 | 35193419 | G | A | -0.0786 | 0.0168 | 2.99E-06 | 0.054 | 0.033 | 0.1 | 21.88903061 |
|  |  | rs72791296 | 5 | 120950050 | C | T | -0.2369 | 0.0466 | 3.78E-07 | -0.088 | 0.078 | 0.26 | 25.84391405 |
|  |  | rs72799710 | 5 | 123161665 | C | T | 0.1014 | 0.0218 | 3.21E-06 | -0.08 | 0.04 | 0.045 | 21.63530006 |
|  |  | rs72828042 | 17 | 33904832 | G | A | 0.3517 | 0.0611 | 3.22E-09 | -0.33 | 0.24 | 0.17 | 33.13311868 |
|  |  | rs72828084 | 17 | 33991650 | C | T | -0.1204 | 0.0225 | 9.25E-08 | -0.049 | 0.044 | 0.27 | 28.63439012 |
|  |  | rs72829264 | 17 | 32832526 | G | A | 0.1601 | 0.0278 | 9.38E-09 | -0.071 | 0.064 | 0.26 | 33.16599814 |
|  |  | rs74810984 | 10 | 129674466 | C | T | -0.2206 | 0.0474 | 1.96E-06 | 0.18 | 0.13 | 0.17 | 21.65979455 |
|  |  | rs75203543 | 3 | 45145171 | C | T | -0.302 | 0.0401 | 4.64E-14 | 0.057 | 0.095 | 0.55 | 56.71855275 |
|  |  | rs76582507 | 9 | 37510072 | G | A | -0.3175 | 0.0677 | 3.26E-06 | -0.27 | 0.15 | 0.065 | 21.99429886 |
|  |  | rs76842834 | 17 | 34883848 | C | T | 0.4206 | 0.0472 | 7.33E-19 | -0.14 | 0.11 | 0.23 | 79.40622307 |
|  |  | rs76960253 | 17 | 34088656 | C | T | -0.5233 | 0.0585 | 5.45E-19 | 0.28 | 0.24 | 0.25 | 80.0183768 |
|  |  | rs79091774 | 3 | 45906878 | C | A | 0.4606 | 0.0751 | 8.83E-10 | -0.047 | 0.091 | 0.6 | 37.61559997 |
|  |  | rs79205588 | 17 | 35223674 | G | A | -0.155 | 0.0313 | 7.62E-07 | -0.14 | 0.08 | 0.08 | 24.52306342 |
|  |  | rs79544064 | 17 | 34092703 | C | A | -0.2442 | 0.0398 | 6.21E-10 | -0.083 | 0.12 | 0.48 | 37.64654933 |
|  |  | rs8074144 | 17 | 35258114 | C | T | -0.0962 | 0.02 | 1.41E-06 | -0.095 | 0.041 | 0.019 | 23.1361 |
|  |  | rs8078470 | 17 | 33774159 | G | A | 0.0966 | 0.0158 | 1.05E-09 | 0.031 | 0.032 | 0.33 | 37.3800673 |
|  |  | rs854222 | 3 | 45397986 | C | A | -0.081 | 0.0177 | 4.46E-06 | -0.055 | 0.038 | 0.15 | 20.94225797 |
|  |  | rs933311 | 17 | 34449405 | C | T | -0.0961 | 0.021 | 4.62E-06 | 0.035 | 0.041 | 0.4 | 20.94151927 |
|  |  | rs9793308 | 1 | 6604585 | G | A | -0.0835 | 0.0178 | 2.52E-06 | -0.04 | 0.042 | 0.33 | 22.00558642 |
|  |  | rs9896155 | 17 | 33794697 | G | A | -0.0826 | 0.0161 | 2.90E-07 | -0.072 | 0.032 | 0.024 | 26.32136106 |
| PDGFbb | 11 | rs113685646 | 2 | 224689693 | G | A | -0.3404 | 0.0557 | 1.01E-09 | 0.37 | 0.19 | 0.054 | 37.34811716 |
|  |  | rs11916118 | 3 | 116912189 | G | A | -0.0889 | 0.0194 | 4.93E-06 | -0.099 | 0.052 | 0.057 | 20.99907004 |
|  |  | rs12990266 | 2 | 224306859 | G | A | -0.2363 | 0.0342 | 3.18E-12 | -0.15 | 0.1 | 0.16 | 47.73921036 |
|  |  | rs181812613 | 2 | 224774551 | G | A | -0.2841 | 0.0381 | 8.26E-14 | 0.14 | 0.16 | 0.39 | 55.6022692 |
|  |  | rs2643354 | 15 | 101970308 | G | A | -0.124 | 0.0261 | 2.09E-06 | 0.099 | 0.061 | 0.11 | 22.57160053 |
|  |  | rs35859699 | 4 | 112184751 | G | A | 0.3952 | 0.0842 | 2.07E-06 | -0.16 | 0.14 | 0.25 | 22.02975609 |
|  |  | rs4965869 | 15 | 101990320 | C | T | -0.184 | 0.0181 | 5.66E-24 | -0.026 | 0.036 | 0.46 | 103.3423888 |
|  |  | rs6051656 | 20 | 374763 | C | T | 0.1288 | 0.0275 | 2.82E-06 | 0.034 | 0.057 | 0.55 | 21.93644959 |
|  |  | rs62027219 | 15 | 101999656 | C | T | 0.1194 | 0.0164 | 3.73E-13 | -0.026 | 0.035 | 0.45 | 53.00550268 |
|  |  | rs72777070 | 2 | 9798877 | G | T | 0.1069 | 0.02 | 8.98E-08 | -0.036 | 0.043 | 0.4 | 28.569025 |
|  |  | rs9936075 | 16 | 7321909 | G | A | 0.0782 | 0.0164 | 1.76E-06 | 0.057 | 0.033 | 0.082 | 22.73661511 |
| RANTES | 9 | rs112072646 | 2 | 53444393 | G | A | -0.4286 | 0.0862 | 6.48E-07 | 0.16 | 0.1 | 0.11 | 24.72235292 |
|  |  | rs147509526 | 19 | 15776330 | C | T | 0.358 | 0.0717 | 6.93E-07 | 0.098 | 0.13 | 0.45 | 24.93031362 |
|  |  | rs62438851 | 6 | 145230309 | G | A | 0.1957 | 0.0414 | 2.33E-06 | -0.032 | 0.049 | 0.51 | 22.34503139 |
|  |  | rs7000423 | 8 | 111053649 | C | T | 0.1318 | 0.0253 | 1.82E-07 | 0.022 | 0.033 | 0.51 | 27.13874611 |
|  |  | rs72793342 | 16 | 30548352 | G | A | 0.1487 | 0.0308 | 1.48E-06 | -0.033 | 0.043 | 0.45 | 23.30883159 |
|  |  | rs74472919 | 13 | 82200650 | C | T | -0.3313 | 0.0605 | 3.97E-08 | -0.13 | 0.088 | 0.14 | 29.98693805 |
|  |  | rs75613039 | 11 | 129576583 | C | T | -0.37 | 0.081 | 4.81E-06 | 0.2 | 0.1 | 0.048 | 20.86572169 |
|  |  | rs818452 | 6 | 152915796 | C | T | -0.2381 | 0.0505 | 2.36E-06 | 0.043 | 0.054 | 0.43 | 22.22982453 |
|  |  | rs9908928 | 17 | 34189017 | G | A | -0.1586 | 0.0319 | 6.23E-07 | 0.021 | 0.04 | 0.6 | 24.71866432 |
| SCF | 5 | rs113127926 | 14 | 98437511 | C | A | -0.1982 | 0.042 | 2.27E-06 | -0.052 | 0.064 | 0.41 | 22.26941043 |
|  |  | rs4841899 | 9 | 137424412 | C | T | 0.1004 | 0.0178 | 1.78E-08 | -0.038 | 0.033 | 0.25 | 31.81466986 |
|  |  | rs635634 | 9 | 136155000 | C | T | 0.1032 | 0.0191 | 6.74E-08 | -0.064 | 0.041 | 0.12 | 29.19393657 |
|  |  | rs78666213 | 4 | 180138649 | G | T | 0.2744 | 0.0576 | 2.59E-06 | -0.11 | 0.11 | 0.31 | 22.69463735 |
|  |  | rs80271436 | 9 | 135897770 | G | A | 0.237 | 0.0485 | 9.95E-07 | -0.14 | 0.083 | 0.093 | 23.87883941 |
| SCGFb | 12 | rs10800456 | 1 | 169520098 | G | A | 0.1566 | 0.0238 | 6.04E-11 | -0.056 | 0.034 | 0.095 | 43.29418826 |
|  |  | rs112346514 | 19 | 12407988 | C | T | 0.3314 | 0.0711 | 2.37E-06 | 0.1 | 0.1 | 0.31 | 21.72530122 |
|  |  | rs116924815 | 19 | 51230733 | C | T | -0.6079 | 0.0738 | 1.74E-16 | 0.1 | 0.12 | 0.38 | 67.85026733 |
|  |  | rs118003677 | 12 | 99755839 | C | T | 0.368 | 0.0786 | 2.81E-06 | -0.099 | 0.14 | 0.48 | 21.9205045 |
|  |  | rs12480722 | 20 | 20228904 | C | T | -0.1624 | 0.0355 | 4.72E-06 | -0.055 | 0.051 | 0.28 | 20.92740329 |
|  |  | rs143829871 | 3 | 47597245 | C | T | 0.1902 | 0.04 | 1.90E-06 | -0.087 | 0.065 | 0.18 | 22.610025 |
|  |  | rs144724875 | 19 | 51195936 | C | T | -0.5459 | 0.084 | 9.19E-11 | 0.16 | 0.13 | 0.23 | 42.23452523 |
|  |  | rs264162 | 18 | 10944026 | G | A | -0.1097 | 0.0234 | 2.69E-06 | 0.016 | 0.031 | 0.61 | 21.97766455 |
|  |  | rs34911860 | 1 | 80350715 | G | A | 0.3675 | 0.0789 | 3.24E-06 | 0.19 | 0.24 | 0.44 | 21.69505125 |
|  |  | rs4737732 | 8 | 66333628 | G | A | 0.1147 | 0.0252 | 4.68E-06 | 0.022 | 0.037 | 0.56 | 20.71694696 |
|  |  | rs62112533 | 19 | 51233758 | C | T | 0.1667 | 0.0281 | 2.57E-09 | -0.075 | 0.04 | 0.059 | 35.19318398 |
|  |  | rs78217154 | 8 | 101554072 | C | T | -0.3997 | 0.0864 | 3.77E-06 | 0.2 | 0.14 | 0.14 | 21.40133236 |
| SDF1a | 8 | rs10474392 | 5 | 91494593 | G | A | -0.0962 | 0.0178 | 1.24E-06 | 0.032 | 0.035 | 0.38 | 29.20855953 |
|  |  | rs12407262 | 1 | 63820276 | G | A | -0.1179 | 0.0266 | 3.99E-06 | -0.1 | 0.044 | 0.019 | 19.64555656 |
|  |  | rs13400104 | 2 | 233810199 | G | A | 0.0647 | 0.0189 | 4.53E-06 | -0.038 | 0.045 | 0.4 | 11.71884886 |
|  |  | rs139840550 | 9 | 38688622 | G | A | -0.1834 | 0.0549 | 3.79E-06 | -0.11 | 0.11 | 0.32 | 11.15973736 |
|  |  | rs149893336 | 4 | 171232591 | G | A | 0.5034 | 0.1081 | 4.52E-06 | 0.46 | 0.23 | 0.043 | 21.68577935 |
|  |  | rs4581824 | 19 | 9185529 | G | T | 0.0701 | 0.0173 | 3.05E-06 | 0.029 | 0.035 | 0.41 | 16.41889138 |
|  |  | rs482700 | 4 | 116067490 | G | A | 0.0893 | 0.0203 | 1.57E-06 | 0.06 | 0.035 | 0.093 | 19.35133102 |
|  |  | rs67689854 | 16 | 89625227 | C | A | 0.0681 | 0.0195 | 3.07E-06 | 0.081 | 0.055 | 0.15 | 12.19621302 |
| TNFa | 2 | rs10834996 | 11 | 26526901 | G | A | -0.1247 | 0.0258 | 1.33E-06 | -0.03 | 0.034 | 0.38 | 23.36111111 |
|  |  | rs111332265 | 5 | 150393107 | G | A | 0.3766 | 0.0754 | 6.63E-07 | 0.049 | 0.072 | 0.5 | 24.94697775 |
| TNFb | 1 | rs76225863 | 1 | 22653595 | G | A | -0.7742 | 0.123 | 1.08E-10 | 0.073 | 0.098 | 0.45 | 39.61832507 |
| TRAIL | 23 | rs11081739 | 18 | 29583126 | G | A | -0.1411 | 0.0202 | 3.34E-12 | 0.1 | 0.042 | 0.012 | 48.79229977 |
|  |  | rs116467561 | 3 | 172529934 | C | T | 0.2099 | 0.035 | 1.81E-09 | -0.26 | 0.084 | 0.0017 | 35.96572245 |
|  |  | rs11657269 | 17 | 6319784 | G | A | -0.1188 | 0.026 | 4.78E-06 | -0.044 | 0.045 | 0.33 | 20.87786982 |
|  |  | rs11699445 | 20 | 15750790 | G | T | -0.0746 | 0.0161 | 3.27E-06 | -0.025 | 0.032 | 0.43 | 21.46969639 |
|  |  | rs117618570 | 18 | 29008594 | G | T | 0.5294 | 0.0423 | 5.62E-36 | 0.11 | 0.11 | 0.31 | 156.6343969 |
|  |  | rs117637258 | 18 | 29871632 | C | T | -0.354 | 0.0345 | 1.71E-24 | 0.085 | 0.075 | 0.26 | 105.2854442 |
|  |  | rs12607805 | 18 | 29117455 | G | A | 0.174 | 0.0254 | 8.20E-12 | 0.084 | 0.073 | 0.25 | 46.92789386 |
|  |  | rs13185784 | 5 | 179694068 | G | A | -0.0846 | 0.0183 | 3.90E-06 | 0.019 | 0.036 | 0.59 | 21.37167428 |
|  |  | rs146783010 | 11 | 89260213 | G | A | 0.6016 | 0.135 | 4.83E-06 | -0.079 | 0.15 | 0.61 | 19.85857668 |
|  |  | rs146827832 | 3 | 172101065 | C | T | -0.1406 | 0.0292 | 1.44E-06 | 0.07 | 0.061 | 0.24 | 23.18488459 |
|  |  | rs148051545 | 19 | 38602957 | C | T | 0.3921 | 0.0848 | 3.86E-06 | 0.12 | 0.12 | 0.33 | 21.37971837 |
|  |  | rs150207604 | 18 | 29336911 | C | A | 0.3671 | 0.0324 | 1.01E-29 | -0.2 | 0.11 | 0.07 | 128.3744951 |
|  |  | rs192145164 | 3 | 172019007 | C | T | -0.3052 | 0.0407 | 1.12E-13 | 0.33 | 0.27 | 0.21 | 56.23157399 |
|  |  | rs1943552 | 18 | 28264855 | G | A | 0.0816 | 0.0171 | 1.85E-06 | -0.05 | 0.033 | 0.13 | 22.77131425 |
|  |  | rs3136594 | 3 | 172230584 | G | A | 0.1361 | 0.0171 | 1.88E-15 | 0.057 | 0.036 | 0.11 | 63.34670497 |
|  |  | rs57396456 | 18 | 27945877 | C | T | 0.5626 | 0.0518 | 1.25E-27 | -0.12 | 0.1 | 0.23 | 117.9614049 |
|  |  | rs62093514 | 18 | 29230977 | C | T | -1.0618 | 0.0552 | 6.86E-82 | 0.18 | 0.11 | 0.11 | 370.004739 |
|  |  | rs62093947 | 18 | 29660305 | C | T | 0.7596 | 0.046 | 3.31E-61 | 0.15 | 0.11 | 0.18 | 272.6806049 |
|  |  | rs6764884 | 3 | 172253678 | G | T | 0.1297 | 0.0227 | 9.96E-09 | -0.026 | 0.046 | 0.57 | 32.64586932 |
|  |  | rs76100852 | 18 | 29404330 | C | T | -0.4336 | 0.0369 | 7.72E-32 | 0.1 | 0.067 | 0.12 | 138.0784219 |
|  |  | rs79287178 | 3 | 172294500 | G | A | 0.4317 | 0.0421 | 9.12E-25 | 0.13 | 0.11 | 0.24 | 105.1477311 |
|  |  | rs9849338 | 3 | 172126476 | C | A | 0.0797 | 0.0161 | 7.65E-07 | -0.029 | 0.033 | 0.38 | 24.50557463 |
|  |  | rs9952273 | 18 | 29575063 | C | T | -0.864 | 0.0499 | 3.86E-69 | 0.41 | 0.14 | 0.0033 | 299.7963864 |
| VEGF | 22 | rs10153304 | 17 | 7721931 | G | A | -0.1547 | 0.0325 | 1.94E-06 | 0.053 | 0.053 | 0.32 | 22.6576 |
|  |  | rs10934631 | 3 | 122697600 | C | T | 0.1151 | 0.0245 | 2.47E-06 | 0.046 | 0.043 | 0.27 | 22.07082049 |
|  |  | rs10967186 | 9 | 2617099 | C | T | -0.0898 | 0.017 | 1.23E-07 | 0.039 | 0.031 | 0.21 | 27.9032526 |
|  |  | rs111889150 | 6 | 43946177 | G | A | 0.1829 | 0.0385 | 1.82E-06 | -0.12 | 0.1 | 0.24 | 22.56866925 |
|  |  | rs1128930 | 6 | 44200325 | C | A | 0.0975 | 0.0177 | 3.88E-08 | -0.024 | 0.035 | 0.49 | 30.34329216 |
|  |  | rs114773511 | 6 | 43796181 | C | T | -0.2186 | 0.0442 | 1.25E-06 | -0.12 | 0.15 | 0.42 | 24.45996192 |
|  |  | rs143479231 | 3 | 193110794 | G | A | 0.2598 | 0.0491 | 1.90E-07 | -0.2 | 0.11 | 0.061 | 27.99724574 |
|  |  | rs1950506 | 6 | 44024262 | G | A | -0.1034 | 0.0179 | 1.02E-08 | 0.043 | 0.034 | 0.2 | 33.36837177 |
|  |  | rs3025020 | 6 | 43749110 | C | T | 0.1254 | 0.0254 | 8.01E-07 | -0.027 | 0.044 | 0.53 | 24.37404675 |
|  |  | rs34695297 | 6 | 43894767 | C | T | 0.1937 | 0.0402 | 1.30E-06 | 0.036 | 0.055 | 0.52 | 23.21705527 |
|  |  | rs34881325 | 9 | 2622134 | C | T | 0.1082 | 0.0189 | 1.04E-08 | -0.064 | 0.037 | 0.081 | 32.77411047 |
|  |  | rs4507572 | 6 | 44135095 | C | T | -0.1007 | 0.0171 | 3.34E-09 | 0.022 | 0.033 | 0.5 | 34.67901235 |
|  |  | rs4523085 | 6 | 43950169 | G | A | 0.0846 | 0.0173 | 1.07E-06 | 0.023 | 0.033 | 0.48 | 23.91379598 |
|  |  | rs56071907 | 16 | 88546405 | C | T | -0.1255 | 0.0271 | 3.92E-06 | 0.061 | 0.054 | 0.25 | 21.44612682 |
|  |  | rs60987108 | 6 | 44427217 | G | A | -0.1852 | 0.0392 | 2.95E-06 | -0.11 | 0.069 | 0.12 | 22.32080383 |
|  |  | rs62401205 | 6 | 43855489 | C | A | 0.2039 | 0.0412 | 1.77E-06 | -0.16 | 0.14 | 0.26 | 24.49288929 |
|  |  | rs6475939 | 9 | 2687860 | C | T | 0.1192 | 0.0182 | 6.64E-11 | 0.023 | 0.034 | 0.5 | 42.8953025 |
|  |  | rs73418461 | 10 | 120222996 | G | A | 0.2492 | 0.0521 | 1.68E-06 | 0.12 | 0.067 | 0.084 | 22.87813558 |
|  |  | rs7919685 | 10 | 65315800 | G | T | 0.0803 | 0.0169 | 2.08E-06 | -0.048 | 0.031 | 0.12 | 22.57655544 |
|  |  | rs9367182 | 6 | 43979345 | C | T | 0.1746 | 0.0283 | 4.44E-10 | -0.029 | 0.057 | 0.61 | 38.06410368 |
|  |  | rs9472141 | 6 | 43818724 | C | T | -0.0819 | 0.0175 | 2.93E-06 | -0.042 | 0.034 | 0.22 | 21.9024 |
|  |  | rs9472176 | 6 | 43934795 | C | T | 0.1498 | 0.0258 | 6.11E-08 | -0.062 | 0.046 | 0.18 | 33.71197644 |

SNP, single nucleotide polymorphism; Beta, beta coefficient; SE, standard error; bNGF, beta-nerve growth factor; CTACK, cutaneous T-cell attracting chemokine; FGFBasic, Fibroblast growth factor basic; GCSF, Granulocyte-colony stimulating factor; GROa, Growth-regulated protein alpha; HGF, Hepatocyte growth factor; IFNg, Interferon gamma; IP10, Interferon gamma-induced protein 10; IL-1b, Interleukin-1-beta; IL1ra, Interleukin-1-receptor antagonist; IL-2, Interleukin-2; IL2ra, Interleukin-2 receptor antagonist; IL-4, Interleukin-4; IL-5, Interleukin-5; IL-6, Interleukin-6; IL-7, Interleukin-7; IL-8, Interleukin-8; IL-9, Interleukin-9; IL-10, Interleukin-10; IL-12p70, Interleukin-12p70; IL-13, Interleukin-13; IL-16, Interleukin-16; IL-17, Interleukin-17; IL-18, Interleukin-18; MCSF, Macrophage colony stimulating factor; MIP1a, Macrophage inflammatory protein 1a; MIP1b, Macrophage inflammatory protein 1b; MIF, Macrophage Migration Inhibitory Factor; MCP1, Monocyte chemoattractant protein-1; MCP3, Monocyte chemoattractant protein-3; MIG, Monokine induced by gamma interferon; PDGFbb, Platelet-derived growth factor BB; RANTES, regulated on Activation, Normal T Cell Expressed and Secreted; SCF, Stem cell factor; SCGFb, Stem cell growth factor beta; SDF1a, Stromal-cell-derived factor 1 alpha; TRAIL, TNF-related apoptosis-inducing ligand; TNFa, Tumor necrosis factor alpha; TNFb, Tumor necrosis factor beta; VEGF, Vascular endothelial growth factor.
