# Supplementary material for: The Association Between Genetically Predicted Systemic Inflammatory Regulators and Polycystic Ovary Syndrome: A Mendelian Randomization Study
Source: Front Endocrinol (Lausanne). 2021 Sep 27;12:731569. doi: 10.3389/fendo.2021.731569 (PMC8503255; doi:10.3389/fendo.2021.731569)
Supplement: Supplementary file 1 [file DataSheet_1.zip › Data Sheet 1/supplementary materials/08_data(supplementary tableS1).docx]

**Supplementary Table S1. Characteristics of the genetic instrument variables for the systemic inflammatory regulators in the Mendelian randomization study at the genome-wide significance level (P < 5 × 10^–8^).**

| **Systematic inflammatory regulators** | **Number of SNPs** | **SNP** | **chr** | **pos** | **Effect allele** | **Other allele** | **Beta of exposure** | **SE of exposure** | **P of exposure** | **Beta of outcome** | **SE of outcome** | **P of outcome** | **F statistics** |
| --- | --- | --- | --- | --- | --- | --- | --- | --- | --- | --- | --- | --- | --- |
| CTACK | 3 | rs2070074 | 9 | 34649442 | G | A | -0.4467 | 0.0374 | 1.79E-32 | -0.037 | 0.054 | 0.48 | 142.6555592 |
|  |  | rs55764737 | 15 | 61323414 | C | T | -0.5313 | 0.0972 | 4.62E-08 | -0.046 | 0.085 | 0.58 | 29.87769585 |
|  |  | rs867811 | 9 | 34663798 | C | T | -0.1908 | 0.026 | 2.37E-13 | 0.032 | 0.032 | 0.32 | 53.85301775 |
| GROa | 4 | rs11265204 | 1 | 159439913 | G | A | 0.1358 | 0.024 | 9.79E-18 | -0.024 | 0.031 | 0.44 | 32.01673611 |
|  |  | rs12727188 | 1 | 159486328 | G | T | 0.2159 | 0.0359 | 3.40E-12 | -0.035 | 0.052 | 0.51 | 36.1673249 |
|  |  | rs3117602 | 4 | 74732886 | C | A | -0.56 | 0.0725 | 5.72E-38 | -0.045 | 0.083 | 0.58 | 59.66230678 |
|  |  | rs7550207 | 1 | 159174885 | C | T | 0.1689 | 0.0284 | 7.70E-11 | 0.028 | 0.037 | 0.44 | 35.36898681 |
| HGF | 2 | rs111548934 | 7 | 81530895 | C | T | 0.2333 | 0.0414 | 1.12E-15 | 0.073 | 0.055 | 0.18 | 31.75621952 |
|  |  | rs3752441 | 4 | 3446820 | C | T | -0.143 | 0.0225 | 2.75E-14 | 0.041 | 0.044 | 0.34 | 40.39308642 |
| IL2ra | 1 | rs34507893 | 10 | 6124598 | G | A | -0.7344 | 0.1164 | 2.91E-10 | -0.44 | 0.26 | 0.088 | 39.8069933 |
| IL-12p70 | 1 | rs10733789 | 10 | 64948684 | C | T | 0.0943 | 0.0172 | 4.03E-08 | -0.033 | 0.034 | 0.33 | 30.05844375 |
| IL-16 | 1 | rs2731672 | 5 | 176842474 | C | T | -0.1683 | 0.0271 | 5.91E-15 | -0.031 | 0.036 | 0.39 | 38.56822483 |
| IL-18 | 3 | rs116656892 | 5 | 68186028 | C | T | -0.5298 | 0.0925 | 1.05E-08 | 0.13 | 0.1 | 0.22 | 32.80503024 |
|  |  | rs141091241 | 11 | 112111460 | C | T | 0.4122 | 0.0728 | 1.83E-08 | 0.13 | 0.096 | 0.17 | 32.05923952 |
|  |  | rs17229943 | 5 | 68682536 | C | A | 0.312 | 0.0463 | 1.62E-11 | 0.072 | 0.082 | 0.38 | 45.40955082 |
| IP10 | 1 | rs9450351 | 6 | 86624320 | C | T | 0.2768 | 0.0489 | 1.48E-08 | 0.047 | 0.062 | 0.45 | 32.0416191 |
| MCP1 | 3 | rs116425179 | 3 | 45598703 | G | A | 0.1476 | 0.0255 | 5.68E-09 | -0.058 | 0.07 | 0.41 | 33.50366782 |
|  |  | rs35333710 | 1 | 159172854 | G | A | -0.1476 | 0.0268 | 3.71E-08 | -0.074 | 0.055 | 0.18 | 30.33214524 |
|  |  | rs6692378 | 1 | 159339241 | C | T | -0.0916 | 0.0158 | 7.31E-09 | 0.02 | 0.032 | 0.54 | 33.61063932 |
| MIP1b | 32 | rs11080371 | 17 | 34375136 | C | T | -0.1035 | 0.0164 | 3.20E-10 | 0.03 | 0.034 | 0.38 | 39.82841315 |
|  |  | rs113010081 | 3 | 46457412 | C | T | 0.5954 | 0.0236 | 3.85E-140 | 0.12 | 0.051 | 0.014 | 636.4930336 |
|  |  | rs113699401 | 17 | 33668796 | G | A | -0.1474 | 0.0212 | 3.35E-12 | -0.033 | 0.049 | 0.5 | 48.34184763 |
|  |  | rs114164513 | 3 | 45272343 | C | T | 0.3269 | 0.0351 | 2.09E-20 | -0.09 | 0.092 | 0.33 | 86.73923913 |
|  |  | rs11651172 | 17 | 34270288 | G | A | 0.1366 | 0.0245 | 2.11E-08 | -0.1 | 0.051 | 0.041 | 31.08631404 |
|  |  | rs117620244 | 17 | 33648381 | C | T | 0.3528 | 0.0495 | 1.87E-12 | -0.1 | 0.16 | 0.52 | 50.79801653 |
|  |  | rs1252860 | 17 | 35125060 | G | A | -0.1232 | 0.0176 | 2.60E-12 | 0.024 | 0.035 | 0.5 | 49 |
|  |  | rs12601380 | 17 | 34904985 | C | A | 0.2159 | 0.0163 | 4.21E-40 | -0.018 | 0.032 | 0.57 | 175.4405887 |
|  |  | rs148561432 | 17 | 33831939 | G | A | 0.2703 | 0.0409 | 6.78E-11 | 0.074 | 0.097 | 0.45 | 43.67626329 |
|  |  | rs148883658 | 17 | 34992469 | C | A | 0.5294 | 0.046 | 1.62E-30 | -0.17 | 0.25 | 0.51 | 132.4500756 |
|  |  | rs150641077 | 17 | 34195451 | G | A | 0.2991 | 0.0492 | 1.25E-09 | 0.28 | 0.17 | 0.096 | 36.95750297 |
|  |  | rs1564708 | 17 | 34825482 | C | T | 0.1744 | 0.0188 | 2.87E-20 | -0.059 | 0.047 | 0.21 | 86.05522861 |
|  |  | rs17693183 | 17 | 34964290 | G | A | 0.5795 | 0.0795 | 8.93E-13 | -0.19 | 0.2 | 0.35 | 53.13401369 |
|  |  | rs1979671 | 3 | 46274215 | C | T | -0.1216 | 0.017 | 7.86E-13 | -0.053 | 0.033 | 0.11 | 51.16456747 |
|  |  | rs1994089 | 17 | 33516594 | C | T | 0.1116 | 0.0162 | 5.50E-12 | -0.036 | 0.032 | 0.26 | 47.45679012 |
|  |  | rs2411190 | 17 | 34992830 | G | A | -0.1822 | 0.0207 | 2.78E-18 | -0.055 | 0.042 | 0.19 | 77.4740134 |
|  |  | rs2531742 | 3 | 45841068 | G | A | -0.1922 | 0.0161 | 1.02E-32 | 0.021 | 0.033 | 0.51 | 142.5131746 |
|  |  | rs2673050 | 3 | 45739807 | G | T | -0.1314 | 0.0161 | 3.14E-16 | -0.018 | 0.031 | 0.57 | 66.60993017 |
|  |  | rs4333119 | 3 | 46096501 | G | T | -0.1444 | 0.0159 | 1.26E-19 | -0.034 | 0.032 | 0.29 | 82.47838298 |
|  |  | rs4795162 | 17 | 35236530 | G | A | -0.1261 | 0.0158 | 1.14E-15 | 0.046 | 0.031 | 0.14 | 63.69656305 |
|  |  | rs55771110 | 3 | 47346717 | G | A | -0.1114 | 0.019 | 4.40E-09 | 0.06 | 0.045 | 0.18 | 34.3766205 |
|  |  | rs60516659 | 17 | 34403297 | G | A | -0.2691 | 0.0248 | 3.64E-27 | 0.063 | 0.053 | 0.23 | 117.7400007 |
|  |  | rs62243190 | 3 | 45524938 | C | T | -0.4572 | 0.0409 | 4.17E-29 | 0.45 | 0.31 | 0.15 | 124.9585069 |
|  |  | rs6505501 | 17 | 34347238 | C | T | 0.1556 | 0.0191 | 3.71E-16 | 0.047 | 0.043 | 0.27 | 66.36704038 |
|  |  | rs72828042 | 17 | 33904832 | G | A | 0.3517 | 0.0611 | 3.22E-09 | -0.33 | 0.24 | 0.17 | 33.13311868 |
|  |  | rs72829264 | 17 | 32832526 | G | A | 0.1601 | 0.0278 | 9.38E-09 | -0.071 | 0.064 | 0.26 | 33.16599814 |
|  |  | rs75203543 | 3 | 45145171 | C | T | -0.302 | 0.0401 | 4.64E-14 | 0.057 | 0.095 | 0.55 | 56.71855275 |
|  |  | rs76842834 | 17 | 34883848 | C | T | 0.4206 | 0.0472 | 7.33E-19 | -0.14 | 0.11 | 0.23 | 79.40622307 |
|  |  | rs76960253 | 17 | 34088656 | C | T | -0.5233 | 0.0585 | 5.45E-19 | 0.28 | 0.24 | 0.25 | 80.0183768 |
|  |  | rs79091774 | 3 | 45906878 | C | A | 0.4606 | 0.0751 | 8.83E-10 | -0.047 | 0.091 | 0.6 | 37.61559997 |
|  |  | rs79544064 | 17 | 34092703 | C | A | -0.2442 | 0.0398 | 6.21E-10 | -0.083 | 0.12 | 0.48 | 37.64654933 |
|  |  | rs8078470 | 17 | 33774159 | G | A | 0.0966 | 0.0158 | 1.05E-09 | 0.031 | 0.032 | 0.33 | 37.3800673 |
| PDGFbb | 5 | rs113685646 | 2 | 224689693 | G | A | -0.3404 | 0.0557 | 1.01E-09 | 0.37 | 0.19 | 0.054 | 37.34811716 |
|  |  | rs12990266 | 2 | 224306859 | G | A | -0.2363 | 0.0342 | 3.18E-12 | -0.15 | 0.1 | 0.16 | 47.73921036 |
|  |  | rs181812613 | 2 | 224774551 | G | A | -0.2841 | 0.0381 | 8.26E-14 | 0.14 | 0.16 | 0.39 | 55.6022692 |
|  |  | rs4965869 | 15 | 101990320 | C | T | -0.184 | 0.0181 | 5.66E-24 | -0.026 | 0.036 | 0.46 | 103.3423888 |
|  |  | rs62027219 | 15 | 101999656 | C | T | 0.1194 | 0.0164 | 3.73E-13 | -0.026 | 0.035 | 0.45 | 53.00550268 |
| RANTES | 1 | rs74472919 | 13 | 82200650 | C | T | -0.3313 | 0.0605 | 2.57E-08 | -0.13 | 0.088 | 0.14 | 29.98693805 |
| SCF | 1 | rs10745373 | 9 | 137424784 | C | T | 0.0992 | 0.0179 | 1.75E-08 | -0.038 | 0.033 | 0.25 | 30.71264942 |
| SCGFb | 4 | rs116924815 | 19 | 51230733 | C | T | -0.6079 | 0.0738 | 1.74E-16 | 0.1 | 0.12 | 0.38 | 67.85026733 |
|  |  | rs144724875 | 19 | 51195936 | C | T | -0.5459 | 0.084 | 9.19E-11 | 0.16 | 0.13 | 0.23 | 42.23452523 |
|  |  | rs17876031 | 5 | 176831119 | G | A | 0.1514 | 0.0255 | 2.25E-09 | -0.044 | 0.033 | 0.19 | 35.25099577 |
|  |  | rs62112533 | 19 | 51233758 | C | T | 0.1667 | 0.0281 | 2.57E-09 | -0.075 | 0.04 | 0.059 | 35.19318398 |
| TNFb | 1 | rs76225863 | 1 | 22653595 | G | A | -0.7742 | 0.123 | 1.08E-10 | 0.073 | 0.098 | 0.45 | 39.61832507 |
| TRAIL | 15 | rs11081739 | 18 | 29583126 | G | A | -0.1411 | 0.0202 | 3.34E-12 | 0.1 | 0.042 | 0.012 | 48.79229977 |
|  |  | rs116467561 | 3 | 172529934 | C | T | 0.2099 | 0.035 | 1.81E-09 | -0.26 | 0.084 | 0.0017 | 35.96572245 |
|  |  | rs117618570 | 18 | 29008594 | G | T | 0.5294 | 0.0423 | 5.62E-36 | 0.11 | 0.11 | 0.31 | 156.6343969 |
|  |  | rs117637258 | 18 | 29871632 | C | T | -0.354 | 0.0345 | 1.71E-24 | 0.085 | 0.075 | 0.26 | 105.2854442 |
|  |  | rs12607805 | 18 | 29117455 | G | A | 0.174 | 0.0254 | 8.20E-12 | 0.084 | 0.073 | 0.25 | 46.92789386 |
|  |  | rs150207604 | 18 | 29336911 | C | A | 0.3671 | 0.0324 | 1.01E-29 | -0.2 | 0.11 | 0.07 | 128.3744951 |
|  |  | rs192145164 | 3 | 172019007 | C | T | -0.3052 | 0.0407 | 1.12E-13 | 0.33 | 0.27 | 0.21 | 56.23157399 |
|  |  | rs3136594 | 3 | 172230584 | G | A | 0.1361 | 0.0171 | 1.88E-15 | 0.057 | 0.036 | 0.11 | 63.34670497 |
|  |  | rs57396456 | 18 | 27945877 | C | T | 0.5626 | 0.0518 | 1.25E-27 | -0.12 | 0.1 | 0.23 | 117.9614049 |
|  |  | rs62093514 | 18 | 29230977 | C | T | -1.0618 | 0.0552 | 6.86E-82 | 0.18 | 0.11 | 0.11 | 370.004739 |
|  |  | rs62093947 | 18 | 29660305 | C | T | 0.7596 | 0.046 | 3.31E-61 | 0.15 | 0.11 | 0.18 | 272.6806049 |
|  |  | rs6764884 | 3 | 172253678 | G | T | 0.1297 | 0.0227 | 9.96E-09 | -0.026 | 0.046 | 0.57 | 32.64586932 |
|  |  | rs76100852 | 18 | 29404330 | C | T | -0.4336 | 0.0369 | 7.72E-32 | 0.1 | 0.067 | 0.12 | 138.0784219 |
|  |  | rs79287178 | 3 | 172294500 | G | A | 0.4317 | 0.0421 | 9.12E-25 | 0.13 | 0.11 | 0.24 | 105.1477311 |
|  |  | rs9952273 | 18 | 29575063 | C | T | -0.864 | 0.0499 | 3.86E-69 | 0.41 | 0.14 | 0.0033 | 299.7963864 |
| VEGF | 9 | rs11538965 | 6 | 44216483 | C | T | -0.24 | 0.041 | 2E-09 | -0.18 | 0.14 | 0.22 | 34.26531826 |
|  |  | rs13209117 | 6 | 44151765 | G | A | -0.1302 | 0.0201 | 5.28E-11 | -0.055 | 0.039 | 0.16 | 41.95945645 |
|  |  | rs1950506 | 6 | 44024262 | G | A | -0.1034 | 0.0179 | 1.02E-08 | 0.043 | 0.034 | 0.2 | 33.36837177 |
|  |  | rs3025007 | 6 | 43747371 | C | T | -0.1022 | 0.0184 | 3.39E-08 | -0.028 | 0.036 | 0.44 | 30.85077977 |
|  |  | rs34881325 | 9 | 2622134 | C | T | 0.1082 | 0.0189 | 1.04E-08 | -0.064 | 0.037 | 0.081 | 32.77411047 |
|  |  | rs41282660 | 6 | 44197006 | G | A | 0.1613 | 0.0263 | 1.33E-09 | -0.057 | 0.057 | 0.32 | 37.61466842 |
|  |  | rs73429788 | 6 | 44142810 | C | T | 0.1715 | 0.0256 | 1.75E-11 | -0.059 | 0.063 | 0.34 | 44.87953186 |
|  |  | rs7757246 | 6 | 44003982 | C | T | -0.1754 | 0.0309 | 2.7E-08 | -0.13 | 0.051 | 0.0093 | 32.22123773 |
|  |  | rs9462957 | 6 | 44025275 | G | A | 0.1166 | 0.0204 | 1.82E-08 | 0.047 | 0.038 | 0.21 | 32.66906959 |

SNP, single nucleotide polymorphism; Beta, beta coefficient; SE, standard error; CTACK, cutaneous T-cell attracting chemokine; GROa, Growth-regulated protein alpha; HGF, Hepatocyte growth factor; IP10, Interferon gamma-induced protein 10; IL1ra, Interleukin-1-receptor antagonist; IL-12p70, Interleukin-12p70; IL-16, Interleukin-16; IL-18, Interleukin-18; MIP1b, Macrophage inflammatory protein 1b; MCP1, Monocyte chemoattractant protein-1; PDGFbb, Platelet-derived growth factor BB; RANTES, regulated on Activation, Normal T Cell Expressed and Secreted; SCF, Stem cell factor; SCGFb, Stem cell growth factor beta; TRAIL, TNF-related apoptosis-inducing ligand; TNFb, Tumor necrosis factor beta; VEGF, Vascular endothelial growth factor.
