# Supplementary material for: The Association Between Genetically Predicted Systemic Inflammatory Regulators and Polycystic Ovary Syndrome: A Mendelian Randomization Study
Source: Front Endocrinol (Lausanne). 2021 Sep 27;12:731569. doi: 10.3389/fendo.2021.731569 (PMC8503255; doi:10.3389/fendo.2021.731569)
Supplement: Supplementary file 1 [file DataSheet_1.zip › Data Sheet 1/supplementary materials/PCOS SNP (supplementary tableS3).docx]

**Supplementary Table S3. SNPs used as instrument variables for PCOS.**

| **Chr** | **Position** | **SNP** | **Effect Allele** | **Other Allele** | **EAF** | **Beta** | **SE** | **Gene** | **P value** | **F statistics** |
| --- | --- | --- | --- | --- | --- | --- | --- | --- | --- | --- |
| 2 | 43561780 | rs7563201 | A | G | 0.4507 | -0.1081 | 0.0172 | THADA | 3.68E-10 | 39.49976 |
| 2 | 213391766 | rs2178575 | A | G | 0.1512 | 0.1663 | 0.0219 | ERBB4 | 3.34E-14 | 57.66287 |
| 5 | 131813204 | rs13164856 | T | C | 0.7291 | 0.1235 | 0.0193 | IRF1/RAD50 | 1.45E-10 | 40.94674 |
| 9 | 5440589 | rs10739076 | A | C | 0.3078 | 0.1097 | 0.0197 | PLGRKT | 2.51E-08 | 31.0085 |
| 9 | 97723266 | rs7864171 | A | G | 0.4284 | -0.0933 | 0.0168 | FANCC | 2.95E-08 | 30.84216 |
| 9 | 126619233 | rs9696009 | A | G | 0.0679 | 0.202 | 0.0311 | DENND1A | 7.96E-11 | 42.18732 |
| 11 | 30226356 | rs11031005 | T | C | 0.8537 | -0.1593 | 0.0223 | ARL14EP/FSHB | 8.66E-13 | 51.02956 |
| 11 | 102043240 | rs11225154 | A | G | 0.0941 | 0.1787 | 0.0272 | YAP1 | 5.44E-11 | 43.16297 |
| 11 | 113949232 | rs1784692 | T | C | 0.8237 | 0.1438 | 0.0226 | ZBTB16 | 1.88E-10 | 40.48563 |
| 12 | 75941042 | rs1795379 | T | C | 0.2398 | -0.1174 | 0.0195 | KRR1 | 1.81E-09 | 36.24657 |

Chr, chromosome; SNP, single nucleotide polymorphism; EAF, effect allele frequency; SE, standard error
