# Supplementary material for: The Association Between Genetically Predicted Systemic Inflammatory Regulators and Polycystic Ovary Syndrome: A Mendelian Randomization Study
Source: Front Endocrinol (Lausanne). 2021 Sep 27;12:731569. doi: 10.3389/fendo.2021.731569 (PMC8503255; doi:10.3389/fendo.2021.731569)
Supplement: Supplementary file 1 [file DataSheet_1.zip › Data Sheet 1/supplementary materials/Supplementary Table S4.docx]

**Supplementary Table S4. MR analysis of 17 systemic inflammatory regulators and PCOS risk.**

| **systematic inflammatory regulators** | | **Number of SNPs** | **OR (95% CI)** | **P** | **P for heterogeneity test** | **P for MR-Egger intercept** |
| --- | --- | --- | --- | --- | --- | --- |
| Interleukin-18 levels | |  |  |  |  |  |
|  | MR Egger | 3 | 0.416 ( 0.091 - 1.903 ) | 0.461043956 | 0.190503183 | 0.440461691 |
|  | Weighted median | 3 | 1.131 ( 0.836 - 1.530 ) | 0.424105611 |  |  |
|  | Inverse variance weighted | 3 | 1.038 ( 0.721 - 1.494 ) | 0.841354399 | 0.121821719 |  |
|  | Simple mode | 3 | 1.315 ( 0.796 - 2.173 ) | 0.39728798 |  |  |
|  | Weighted mode | 3 | 1.321 ( 0.757 - 2.304 ) | 0.430427038 |  |  |
| Macrophage inflammatory protein 1b levels | |  |  |  |  |  |
|  | MR Egger | 32 | 1.055 ( 0.862 - 1.292 ) | 0.605678909 | 0.067518626 | 0.446849894 |
|  | Weighted median | 32 | 0.989 ( 0.855 - 1.145 ) | 0.883504962 |  |  |
|  | Inverse variance weighted | 32 | 0.985 ( 0.891 - 1.089 ) | 0.76716872 | 0.072370575 |  |
|  | Simple mode | 32 | 0.755 ( 0.554 - 1.029 ) | 0.085395922 |  |  |
|  | Weighted mode | 32 | 1.169 ( 0.996 - 1.372 ) | 0.06475669 |  |  |
| Interleukin-12p70 levels | |  |  |  |  |  |
|  | Wald ratio | 1 | 0.705 ( 0.348 - 1.429 ) | 0.331753367 |  |  |
| Growth-regulated protein alpha levels | |  |  |  |  |  |
|  | MR Egger | 4 | 1.135 ( 0.757 - 1.700 ) | 0.603070482 | 0.474090476 | 0.584235341 |
|  | Weighted median | 4 | 1.021 ( 0.812 - 1.283 ) | 0.859812335 |  |  |
|  | Inverse variance weighted | 4 | 1.009 ( 0.832 - 1.224 ) | 0.927189508 | 0.591149613 |  |
|  | Simple mode | 4 | 0.864 ( 0.618 - 1.208 ) | 0.455811986 |  |  |
|  | Weighted mode | 4 | 1.094 ( 0.825 - 1.452 ) | 0.577300569 |  |  |
| Interleukin-16 levels | |  |  |  |  |  |
|  | Wald ratio | 1 | 1.202 ( 0.791 - 1.828 ) | 0.38917685 |  |  |
| RANTES levels | |  |  |  |  |  |
|  | Wald ratio | 1 | 1.481 ( 0.880 - 2.492 ) | 0.139602546 |  |  |
| CTACK levels | |  |  |  |  |  |
|  | MR Egger | 3 | 1.286 ( 0.876 - 1.888 ) | 0.421579118 | 0.901684846 | 0.418303342 |
|  | Weighted median | 3 | 1.087 ( 0.899 - 1.313 ) | 0.389733582 |  |  |
|  | Inverse variance weighted | 3 | 1.022 ( 0.867 - 1.204 ) | 0.796252378 | 0.428338429 |  |
|  | Simple mode | 3 | 1.088 ( 0.841 - 1.407 ) | 0.585716321 |  |  |
|  | Weighted mode | 3 | 1.088 ( 0.874 - 1.354 ) | 0.53094713 |  |  |
| Platelet-derived growth factor BB levels | |  |  |  |  |  |
|  | MR Egger | 5 | 0.991 ( 0.197 - 4.978 ) | 0.991493597 | 0.048580983 | 0.997514609 |
|  | Weighted median | 5 | 1.025 ( 0.742 - 1.415 ) | 0.88204079 |  |  |
|  | Inverse variance weighted | 5 | 0.993 ( 0.672 - 1.467 ) | 0.972518402 | 0.096113545 |  |
|  | Simple mode | 5 | 0.819 ( 0.470 - 1.426 ) | 0.518799009 |  |  |
|  | Weighted mode | 5 | 1.062 ( 0.730 - 1.546 ) | 0.76710087 |  |  |
| Hepatocyte growth factor levels | |  |  |  |  |  |
|  | Inverse variance weighted | 2 | 1.095 ( 0.621 - 1.932 ) | 0.752966032 | 0.121886755 |  |
| Stem cell growth factor beta levels | |  |  |  |  |  |
|  | MR Egger | 4 | 0.844 ( 0.552 - 1.290 ) | 0.515721016 | 0.794952383 | 0.596993844 |
|  | Weighted median | 4 | 0.747 ( 0.576 - 0.970 ) | 0.02871235 |  |  |
|  | Inverse variance weighted | 4 | 0.752 ( 0.605 - 0.934 ) | 0.009882917 | 0.838252889 |  |
|  | Simple mode | 4 | 0.753 ( 0.546 - 1.038 ) | 0.181425635 |  |  |
|  | Weighted mode | 4 | 0.759 ( 0.551 - 1.044 ) | 0.188660833 |  |  |
| Interleukin-2 receptor antagonist levels | |  |  |  |  |  |
|  | Wald ratio | 1 | 1.821 ( 0.910 - 3.644 ) | 0.090587322 |  |  |
| TRAIL levels | |  |  |  |  |  |
|  | MR Egger | 15 | 0.870 ( 0.663 - 1.141 ) | 0.332813364 | 0.000392869 | 0.979424931 |
|  | Weighted median | 15 | 0.828 ( 0.708 - 0.968 ) | 0.018018756 |  |  |
|  | Inverse variance weighted | 15 | 0.867 ( 0.735 - 1.024 ) | 0.092440631 | 0.000700361 |  |
|  | Simple mode | 15 | 0.810 ( 0.599 - 1.095 ) | 0.191484246 |  |  |
|  | Weighted mode | 15 | 0.836 ( 0.696 - 1.006 ) | 0.078188228 |  |  |
| Tumor necrosis factor beta levels | |  |  |  |  |  |
|  | Wald ratio | 1 | 0.910 ( 0.710 - 1.166 ) | 0.456333409 |  |  |
| Stem cell factor levels | |  |  |  |  |  |
|  | Wald ratio | 1 | 0.682 ( 0.355 - 1.309 ) | 0.249520366 |  |  |
| Interferon gamma-induced protein 10 levels | |  |  |  |  |  |
|  | Wald ratio | 1 | 1.185 ( 0.764 - 1.838 ) | 0.448412361 |  |  |
| Monocyte chemoattractant protein-1 levels | |  |  |  |  |  |
|  | MR Egger | 3 | 2.179 ( 0.134 - 35.547 ) | 0.68147142 | 0.138135071 | 0.675204684 |
|  | Weighted median | 3 | 0.904 ( 0.519 - 1.574 ) | 0.72242913 |  |  |
|  | Inverse variance weighted | 3 | 1.004 ( 0.592 - 1.703 ) | 0.988886716 | 0.236085385 |  |
|  | Simple mode | 3 | 0.736 ( 0.338 - 1.600 ) | 0.519845215 |  |  |
|  | Weighted mode | 3 | 0.762 ( 0.384 - 1.512 ) | 0.518512621 |  |  |
| Vascular endothelial growth factor levels | |  |  |  |  |  |
|  | MR Egger | 9 | 2.438 ( 0.531 - 11.207 ) | 0.289700468 | 0.029155089 | 0.330030618 |
|  | Weighted median | 9 | 1.321 ( 0.900 - 1.937 ) | 0.154762796 |  |  |
|  | Inverse variance weighted | 9 | 1.101 ( 0.787 - 1.541 ) | 0.573379988 | 0.021016823 |  |
|  | Simple mode | 9 | 1.625 ( 0.710 - 3.716 ) | 0.283308781 |  |  |
|  | Weighted mode | 9 | 1.566 ( 0.696 - 3.524 ) | 0.310368761 |  |  |

Abbreviations: SNP, single nucleotide polymorphism; OR, odds ratio; CI, confidence interval.
