# Supplementary material for: The Association Between Genetically Predicted Systemic Inflammatory Regulators and Polycystic Ovary Syndrome: A Mendelian Randomization Study
Source: Front Endocrinol (Lausanne). 2021 Sep 27;12:731569. doi: 10.3389/fendo.2021.731569 (PMC8503255; doi:10.3389/fendo.2021.731569)
Supplement: Supplementary file 1 [file DataSheet_1.zip › Data Sheet 1/supplementary materials/Supplementary Table S5.docx]

| **systematic inflammatory regulators** | | **Number of SNPs** | **OR (95% CI)** | **P** | **P for heterogeneity test** | **P for MR-Egger intercept** |
| --- | --- | --- | --- | --- | --- | --- |
| Interleukin-7 levels | |  |  |  |  |  |
|  | MR Egger | 8 | 1.071 ( 0.576 - 1.989 ) | 0.836385198 | 0.019795248 | 0.407330279 |
|  | Weighted median | 8 | 0.813 ( 0.637 - 1.037 ) | 0.095684013 |  |  |
|  | Inverse variance weighted | 8 | 0.832 ( 0.633 - 1.093 ) | 0.186919808 | 0.01706534 |  |
|  | Simple mode | 8 | 0.868 ( 0.593 - 1.269 ) | 0.4878263 |  |  |
|  | Weighted mode | 8 | 0.848 ( 0.599 - 1.201 ) | 0.384743789 |  |  |
| Stromal-cell-derived factor 1 alpha levels | |  |  |  |  |  |
|  | MR Egger | 8 | 2.704 ( 0.974 - 7.502 ) | 0.104660426 | 0.208214757 | 0.298983706 |
|  | Weighted median | 8 | 1.879 ( 1.182 - 2.987 ) | 0.007620882 |  |  |
|  | Inverse variance weighted | 8 | 1.563 ( 1.055 - 2.315 ) | 0.025923546 | 0.175028882 |  |
|  | Simple mode | 8 | 2.084 ( 1.015 - 4.282 ) | 0.085696524 |  |  |
|  | Weighted mode | 8 | 2.110 ( 1.007 - 4.421 ) | 0.088451287 |  |  |
| Interleukin-13 levels | |  |  |  |  |  |
|  | MR Egger | 8 | 1.560 ( 1.158 - 2.103 ) | 0.026551941 | 0.372065277 | 0.014323678 |
|  | Weighted median | 8 | 0.829 ( 0.621 - 1.105 ) | 0.200205456 |  |  |
|  | Inverse variance weighted | 8 | 1.039 ( 0.774 - 1.395 ) | 0.797905321 | 0.008114128 |  |
|  | Simple mode | 8 | 0.704 ( 0.469 - 1.058 ) | 0.134895422 |  |  |
|  | Weighted mode | 8 | 0.707 ( 0.438 - 1.140 ) | 0.198269206 |  |  |
| Stem cell factor levels | |  |  |  |  |  |
|  | MR Egger | 5 | 0.913 ( 0.348 - 2.392 ) | 0.864947892 | 0.243842306 | 0.664026252 |
|  | Weighted median | 5 | 0.675 ( 0.449 - 1.014 ) | 0.058237951 |  |  |
|  | Inverse variance weighted | 5 | 0.734 ( 0.528 - 1.021 ) | 0.066478756 | 0.343899858 |  |
|  | Simple mode | 5 | 0.607 ( 0.348 - 1.059 ) | 0.153618603 |  |  |
|  | Weighted mode | 5 | 0.614 ( 0.331 - 1.140 ) | 0.197079397 |  |  |
| Fibroblast growth factor basic levels | |  |  |  |  |  |
|  | MR Egger | 3 | 0.207 ( 0.026 - 1.641 ) | 0.376097808 | 0.644772922 | 0.341196289 |
|  | Weighted median | 3 | 1.279 ( 0.679 - 2.408 ) | 0.446788133 |  |  |
|  | Inverse variance weighted | 3 | 1.167 ( 0.645 - 2.114 ) | 0.609629892 | 0.217911977 |  |
|  | Simple mode | 3 | 1.520 ( 0.614 - 3.765 ) | 0.460945986 |  |  |
|  | Weighted mode | 3 | 1.547 ( 0.583 - 4.102 ) | 0.472976586 |  |  |
| Vascular endothelial growth factor levels | |  |  |  |  |  |
|  | MR Egger | 22 | 1.158 ( 0.659 - 2.033 ) | 0.615927308 | 0.115519226 | 0.372209083 |
|  | Weighted median | 22 | 0.805 ( 0.644 - 1.006 ) | 0.056500928 |  |  |
|  | Inverse variance weighted | 22 | 0.902 ( 0.757 - 1.076 ) | 0.253508091 | 0.116343883 |  |
|  | Simple mode | 22 | 0.652 ( 0.424 - 1.004 ) | 0.065418918 |  |  |
|  | Weighted mode | 22 | 0.695 ( 0.425 - 1.138 ) | 0.162988015 |  |  |
| Macrophage inflammatory protein 1b levels | |  |  |  |  |  |
|  | MR Egger | 67 | 0.961 ( 0.803 - 1.151 ) | 0.666620794 | 0.000741924 | 0.359992851 |
|  | Weighted median | 67 | 1.124 ( 0.991 - 1.275 ) | 0.06894837 |  |  |
|  | Inverse variance weighted | 67 | 1.033 ( 0.939 - 1.136 ) | 0.510086766 | 0.000721842 |  |
|  | Simple mode | 67 | 0.714 ( 0.517 - 0.986 ) | 0.044828246 |  |  |
|  | Weighted mode | 67 | 1.138 ( 0.986 - 1.314 ) | 0.0810399 |  |  |
| Granulocyte-colony stimulating factor levels | |  |  |  |  |  |
|  | MR Egger | 3 | 0.000 ( 0.000 - 2.942 ) | 0.318881526 | 0.425953643 | 0.323578912 |
|  | Weighted median | 3 | 0.728 ( 0.382 - 1.388 ) | 0.334712602 |  |  |
|  | Inverse variance weighted | 3 | 0.769 ( 0.396 - 1.497 ) | 0.44027042 | 0.145445208 |  |
|  | Simple mode | 3 | 0.564 ( 0.229 - 1.388 ) | 0.338828965 |  |  |
|  | Weighted mode | 3 | 0.601 ( 0.238 - 1.521 ) | 0.39496543 |  |  |
| Interleukin-17 levels | |  |  |  |  |  |
|  | Inverse variance weighted | 2 | 1.794 ( 1.150 - 2.801 ) | 0.010038563 | 0.982672942 |  |
| TRAIL levels | |  |  |  |  |  |
|  | MR Egger | 23 | 0.882 ( 0.728 - 1.069 ) | 0.215204186 | 0.001924636 | 0.930165966 |
|  | Weighted median | 23 | 0.832 ( 0.711 - 0.974 ) | 0.02194523 |  |  |
|  | Inverse variance weighted | 23 | 0.877 ( 0.767 - 1.003 ) | 0.054569517 | 0.002924431 |  |
|  | Simple mode | 23 | 0.757 ( 0.550 - 1.042 ) | 0.101320045 |  |  |
|  | Weighted mode | 23 | 0.819 ( 0.692 - 0.969 ) | 0.029348687 |  |  |
| Macrophage Migration Inhibitory Factor levels | |  |  |  |  |  |
|  | Inverse variance weighted | 2 | 1.007 ( 0.661 - 1.536 ) | 0.972509581 | 0.381213672 |  |
| Monokine induced by gamma interferon levels | |  |  |  |  |  |
|  | MR Egger | 10 | 0.888 ( 0.563 - 1.400 ) | 0.622868507 | 0.068126406 | 0.183970305 |
|  | Weighted median | 10 | 1.293 ( 1.012 - 1.652 ) | 0.039768061 |  |  |
|  | Inverse variance weighted | 10 | 1.195 ( 0.949 - 1.504 ) | 0.130058701 | 0.03062286 |  |
|  | Simple mode | 10 | 1.412 ( 0.952 - 2.094 ) | 0.120386392 |  |  |
|  | Weighted mode | 10 | 1.352 ( 1.036 - 1.764 ) | 0.053480106 |  |  |
| Hepatocyte growth factor levels | |  |  |  |  |  |
|  | MR Egger | 8 | 1.529 ( 0.841 - 2.780 ) | 0.213054429 | 0.204154491 | 0.211269337 |
|  | Weighted median | 8 | 1.244 ( 0.901 - 1.719 ) | 0.184523971 |  |  |
|  | Inverse variance weighted | 8 | 1.043 ( 0.787 - 1.380 ) | 0.770796957 | 0.127495802 |  |
|  | Simple mode | 8 | 1.286 ( 0.708 - 2.339 ) | 0.436118458 |  |  |
|  | Weighted mode | 8 | 1.387 ( 0.884 - 2.175 ) | 0.197754426 |  |  |
| Interleukin-2 levels | |  |  |  |  |  |
|  | MR Egger | 7 | 0.748 ( 0.420 - 1.334 ) | 0.370366464 | 0.116354294 | 0.687857457 |
|  | Weighted median | 7 | 0.758 ( 0.571 - 1.006 ) | 0.054924725 |  |  |
|  | Inverse variance weighted | 7 | 0.836 ( 0.653 - 1.071 ) | 0.156482208 | 0.165710066 |  |
|  | Simple mode | 7 | 0.719 ( 0.475 - 1.090 ) | 0.171369443 |  |  |
|  | Weighted mode | 7 | 0.735 ( 0.484 - 1.116 ) | 0.198928528 |  |  |
| Monocyte chemoattractant protein-3 levels | |  |  |  |  |  |
|  | Inverse variance weighted | 2 | 1.056 ( 0.691 - 1.616 ) | 0.800588008 | 0.049116282 |  |
| Tumor necrosis factor beta levels | |  |  |  |  |  |
|  | Wald ratio | 1 | 0.910 ( 0.710 - 1.166 ) | 0.456333409 |  |  |
| Growth-regulated protein alpha levels | |  |  |  |  |  |
|  | MR Egger | 6 | 1.160 ( 0.603 - 2.228 ) | 0.67991364 | 0.512162154 | 0.314322601 |
|  | Weighted median | 6 | 0.851 ( 0.640 - 1.131 ) | 0.265107569 |  |  |
|  | Inverse variance weighted | 6 | 0.809 ( 0.646 - 1.015 ) | 0.067083463 | 0.466415884 |  |
|  | Simple mode | 6 | 0.837 ( 0.582 - 1.203 ) | 0.381289945 |  |  |
|  | Weighted mode | 6 | 0.846 ( 0.601 - 1.191 ) | 0.381864349 |  |  |
| Interleukin-18 levels | |  |  |  |  |  |
|  | MR Egger | 10 | 1.055 ( 0.715 - 1.555 ) | 0.795631894 | 0.122638041 | 0.72450576 |
|  | Weighted median | 10 | 0.947 ( 0.758 - 1.182 ) | 0.627785808 |  |  |
|  | Inverse variance weighted | 10 | 0.991 ( 0.821 - 1.196 ) | 0.92405387 | 0.166698816 |  |
|  | Simple mode | 10 | 0.758 ( 0.469 - 1.226 ) | 0.288517501 |  |  |
|  | Weighted mode | 10 | 0.773 ( 0.500 - 1.193 ) | 0.274693833 |  |  |
| Interferon gamma-induced protein 10 levels | |  |  |  |  |  |
|  | MR Egger | 7 | 0.752 ( 0.425 - 1.330 ) | 0.372380056 | 0.052083799 | 0.382750653 |
|  | Weighted median | 7 | 0.924 ( 0.695 - 1.227 ) | 0.584944072 |  |  |
|  | Inverse variance weighted | 7 | 0.957 ( 0.722 - 1.269 ) | 0.761496921 | 0.043498678 |  |
|  | Simple mode | 7 | 1.328 ( 0.702 - 2.514 ) | 0.416709909 |  |  |
|  | Weighted mode | 7 | 0.701 ( 0.401 - 1.225 ) | 0.25868005 |  |  |
| Interleukin-5 levels | |  |  |  |  |  |
|  | Inverse variance weighted | 2 | 0.647 ( 0.418 - 1.003 ) | 0.051615636 | 0.612938467 |  |
| Interleukin-12p70 levels | |  |  |  |  |  |
|  | MR Egger | 3 | 10.816 ( 0.550 - 212.693 ) | 0.361676038 | 0.341869814 | 0.343220754 |
|  | Weighted median | 3 | 0.735 ( 0.401 - 1.350 ) | 0.321689129 |  |  |
|  | Inverse variance weighted | 3 | 0.886 ( 0.439 - 1.787 ) | 0.735275156 | 0.157435174 |  |
|  | Simple mode | 3 | 0.734 ( 0.378 - 1.425 ) | 0.456803053 |  |  |
|  | Weighted mode | 3 | 0.731 ( 0.397 - 1.346 ) | 0.420377961 |  |  |
| Interleukin-8 levels | |  |  |  |  |  |
|  | MR Egger | 3 | 1.132 ( 0.592 - 2.163 ) | 0.771926578 | 0.057501124 | 0.818886187 |
|  | Weighted median | 3 | 1.089 ( 0.835 - 1.421 ) | 0.528961822 |  |  |
|  | Inverse variance weighted | 3 | 1.050 ( 0.777 - 1.419 ) | 0.750926935 | 0.14110065 |  |
|  | Simple mode | 3 | 1.184 ( 0.828 - 1.695 ) | 0.452626087 |  |  |
|  | Weighted mode | 3 | 1.110 ( 0.846 - 1.454 ) | 0.530021523 |  |  |
| Platelet-derived growth factor BB levels | |  |  |  |  |  |
|  | MR Egger | 11 | 0.631 ( 0.299 - 1.329 ) | 0.256604688 | 0.05951296 | 0.223190068 |
|  | Weighted median | 11 | 0.979 ( 0.715 - 1.340 ) | 0.894470097 |  |  |
|  | Inverse variance weighted | 11 | 0.997 ( 0.736 - 1.349 ) | 0.98408145 | 0.034512762 |  |
|  | Simple mode | 11 | 0.723 ( 0.380 - 1.378 ) | 0.347688272 |  |  |
|  | Weighted mode | 11 | 0.991 ( 0.699 - 1.406 ) | 0.962622952 |  |  |
| Interleukin-1-beta levels | |  |  |  |  |  |
|  | Inverse variance weighted | 2 | 0.627 ( 0.346 - 1.137 ) | 0.124454039 | 0.641523006 |  |
| Stem cell growth factor beta levels | |  |  |  |  |  |
|  | MR Egger | 12 | 0.802 ( 0.566 - 1.138 ) | 0.24458938 | 0.328455029 | 0.783981687 |
|  | Weighted median | 12 | 0.768 ( 0.617 - 0.956 ) | 0.018002159 |  |  |
|  | Inverse variance weighted | 12 | 0.838 ( 0.712 - 0.986 ) | 0.033656824 | 0.404493294 |  |
|  | Simple mode | 12 | 0.719 ( 0.511 - 1.012 ) | 0.085370906 |  |  |
|  | Weighted mode | 12 | 0.741 ( 0.554 - 0.992 ) | 0.068952516 |  |  |
| Monocyte chemoattractant protein-1 levels | |  |  |  |  |  |
|  | MR Egger | 12 | 1.348 ( 0.672 - 2.706 ) | 0.420221861 | 0.307743836 | 0.59511683 |
|  | Weighted median | 12 | 0.977 ( 0.690 - 1.385 ) | 0.896295722 |  |  |
|  | Inverse variance weighted | 12 | 1.123 ( 0.885 - 1.427 ) | 0.339811375 | 0.362015595 |  |
|  | Simple mode | 12 | 0.772 ( 0.393 - 1.517 ) | 0.467963813 |  |  |
|  | Weighted mode | 12 | 0.783 ( 0.413 - 1.484 ) | 0.468815366 |  |  |
| Interleukin-9 levels | |  |  |  |  |  |
|  | MR Egger | 3 | 0.595 ( 0.310 - 1.143 ) | 0.363100372 | 0.989498529 | 0.357990379 |
|  | Weighted median | 3 | 0.869 ( 0.594 - 1.270 ) | 0.46841551 |  |  |
|  | Inverse variance weighted | 3 | 0.949 ( 0.673 - 1.338 ) | 0.764821955 | 0.283923643 |  |
|  | Simple mode | 3 | 0.804 ( 0.487 - 1.326 ) | 0.48280103 |  |  |
|  | Weighted mode | 3 | 0.807 ( 0.521 - 1.249 ) | 0.437511636 |  |  |
| Interleukin-4 levels | |  |  |  |  |  |
|  | MR Egger | 3 | 0.376 ( 0.022 - 6.540 ) | 0.623506284 | 0.400787951 | 0.776480323 |
|  | Weighted median | 3 | 0.722 ( 0.419 - 1.243 ) | 0.24001278 |  |  |
|  | Inverse variance weighted | 3 | 0.637 ( 0.413 - 0.983 ) | 0.041743961 | 0.657006754 |  |
|  | Simple mode | 3 | 0.723 ( 0.373 - 1.404 ) | 0.439329457 |  |  |
|  | Weighted mode | 3 | 0.723 ( 0.395 - 1.325 ) | 0.404460176 |  |  |
| beta-nerve growth factor levels | |  |  |  |  |  |
|  | MR Egger | 5 | 0.460 ( 0.128 - 1.659 ) | 0.320678971 | 0.104789786 | 0.411962862 |
|  | Weighted median | 5 | 0.886 ( 0.618 - 1.269 ) | 0.508851283 |  |  |
|  | Inverse variance weighted | 5 | 0.834 ( 0.578 - 1.204 ) | 0.333037075 | 0.091760751 |  |
|  | Simple mode | 5 | 0.618 ( 0.319 - 1.198 ) | 0.227208174 |  |  |
|  | Weighted mode | 5 | 1.140 ( 0.638 - 2.036 ) | 0.68056808 |  |  |
| RANTES levels | |  |  |  |  |  |
|  | MR Egger | 9 | 0.765 ( 0.451 - 1.299 ) | 0.354153391 | 0.21876792 | 0.552071477 |
|  | Weighted median | 9 | 0.846 ( 0.661 - 1.083 ) | 0.184761365 |  |  |
|  | Inverse variance weighted | 9 | 0.894 ( 0.739 - 1.082 ) | 0.251036068 | 0.263023648 |  |
|  | Simple mode | 9 | 0.815 ( 0.547 - 1.213 ) | 0.342436905 |  |  |
|  | Weighted mode | 9 | 0.821 ( 0.575 - 1.172 ) | 0.309732083 |  |  |
| Macrophage inflammatory protein 1a levels | |  |  |  |  |  |
|  | MR Egger | 5 | 1.840 ( 0.333 - 10.184 ) | 0.534946944 | 0.016449234 | 0.453525119 |
|  | Weighted median | 5 | 0.934 ( 0.622 - 1.401 ) | 0.741239418 |  |  |
|  | Inverse variance weighted | 5 | 0.894 ( 0.572 - 1.397 ) | 0.623352424 | 0.012357266 |  |
|  | Simple mode | 5 | 0.605 ( 0.276 - 1.326 ) | 0.277800818 |  |  |
|  | Weighted mode | 5 | 1.275 ( 0.590 - 2.757 ) | 0.570555823 |  |  |
| Tumor necrosis factor alpha levels | |  |  |  |  |  |
|  | Inverse variance weighted | 2 | 1.181 ( 0.869 - 1.605 ) | 0.287421311 | 0.740096866 |  |
| Eotaxin levels | |  |  |  |  |  |
|  | MR Egger | 15 | 1.071 ( 0.462 - 2.484 ) | 0.875282845 | 0.000678041 | 0.891139626 |
|  | Weighted median | 15 | 1.008 ( 0.744 - 1.365 ) | 0.959804598 |  |  |
|  | Inverse variance weighted | 15 | 1.012 ( 0.768 - 1.335 ) | 0.930028247 | 0.001165185 |  |
|  | Simple mode | 15 | 1.449 ( 0.691 - 3.038 ) | 0.343030589 |  |  |
|  | Weighted mode | 15 | 0.646 ( 0.256 - 1.631 ) | 0.370995278 |  |  |
| Interleukin-10 levels | |  |  |  |  |  |
|  | Inverse variance weighted | 2 | 0.761 ( 0.440 - 1.317 ) | 0.329013671 | 0.785455506 |  |
| Interleukin-16 levels | |  |  |  |  |  |
|  | MR Egger | 7 | 1.062 ( 0.630 - 1.789 ) | 0.829663299 | 0.063443158 | 0.951292182 |
|  | Weighted median | 7 | 1.255 ( 0.978 - 1.612 ) | 0.074586354 |  |  |
|  | Inverse variance weighted | 7 | 1.046 ( 0.829 - 1.321 ) | 0.702276825 | 0.106619506 |  |
|  | Simple mode | 7 | 1.278 ( 0.857 - 1.906 ) | 0.274294617 |  |  |
|  | Weighted mode | 7 | 1.278 ( 0.846 - 1.930 ) | 0.287966157 |  |  |
| CTACK levels | |  |  |  |  |  |
|  | MR Egger | 8 | 1.150 ( 0.768 - 1.721 ) | 0.52207654 | 0.10689305 | 0.655977017 |
|  | Weighted median | 8 | 1.087 ( 0.904 - 1.308 ) | 0.375510786 |  |  |
|  | Inverse variance weighted | 8 | 1.056 ( 0.887 - 1.256 ) | 0.541745022 | 0.146049634 |  |
|  | Simple mode | 8 | 1.157 ( 0.864 - 1.550 ) | 0.359374692 |  |  |
|  | Weighted mode | 8 | 1.067 ( 0.877 - 1.299 ) | 0.535943613 |  |  |
| Interferon gamma levels | |  |  |  |  |  |
|  | MR Egger | 4 | 0.887 ( 0.191 - 4.119 ) | 0.892360009 | 0.069211389 | 0.722226093 |
|  | Weighted median | 4 | 1.331 ( 0.744 - 2.380 ) | 0.334868124 |  |  |
|  | Inverse variance weighted | 4 | 1.185 ( 0.677 - 2.072 ) | 0.552178847 | 0.122405696 |  |
|  | Simple mode | 4 | 1.547 ( 0.637 - 3.754 ) | 0.406130537 |  |  |
|  | Weighted mode | 4 | 1.459 ( 0.659 - 3.227 ) | 0.420180404 |  |  |
| Interleukin-6 levels | |  |  |  |  |  |
|  | Inverse variance weighted | 2 | 1.447 ( 0.863 - 2.425 ) | 0.16073317 | 0.976006437 |  |
| Interleukin-1-receptor antagonist levels | |  |  |  |  |  |
|  | Inverse variance weighted | 2 | 0.746 ( 0.489 - 1.139 ) | 0.174432738 | 0.579445444 |  |
| Interleukin-2 receptor antagonist levels | |  |  |  |  |  |
|  | MR Egger | 6 | 1.345 ( 0.559 - 3.238 ) | 0.544184437 | 0.037670609 | 0.33034991 |
|  | Weighted median | 6 | 0.730 ( 0.531 - 1.004 ) | 0.052872011 |  |  |
|  | Inverse variance weighted | 6 | 0.859 ( 0.585 - 1.261 ) | 0.438588708 | 0.020847432 |  |
|  | Simple mode | 6 | 0.687 ( 0.408 - 1.156 ) | 0.216768316 |  |  |
|  | Weighted mode | 6 | 0.695 ( 0.446 - 1.082 ) | 0.168266319 |  |  |
| Macrophage colony stimulating factor levels | |  |  |  |  |  |
|  | Inverse variance weighted | 2 | 1.381 ( 0.943 - 2.023 ) | 0.097065905 | 0.461746901 |  |
