# Supplementary material for: The Association Between Genetically Predicted Systemic Inflammatory Regulators and Polycystic Ovary Syndrome: A Mendelian Randomization Study
Source: Front Endocrinol (Lausanne). 2021 Sep 27;12:731569. doi: 10.3389/fendo.2021.731569 (PMC8503255; doi:10.3389/fendo.2021.731569)
Supplement: Supplementary file 1 [file DataSheet_1.zip › Data Sheet 1/supplementary materials/Supplementary Table S6.docx]

**Supplementary Table S6. MR analysis of the association between PCOS and 41 systemic inflammatory regulators.**

| **systematic inflammatory regulators** | | **Number of SNPs** | **OR (95% CI)** | **P** | **P for heterogeneity test** | **P for MR-Egger intercept** |
| --- | --- | --- | --- | --- | --- | --- |
| Interleukin-10 levels | |  |  |  |  |  |
|  | MR Egger | 10 | 1.044 ( 0.528 - 2.061 ) | 0.905230686 | 0.012569239 | 0.925385163 |
|  | Weighted median | 10 | 1.027 ( 0.890 - 1.186 ) | 0.713469124 |  |  |
|  | Inverse variance weighted | 10 | 1.078 ( 0.936 - 1.242 ) | 0.295647604 | 0.021363714 |  |
|  | Simple mode | 10 | 0.919 ( 0.685 - 1.233 ) | 0.587676421 |  |  |
|  | Weighted mode | 10 | 0.945 ( 0.728 - 1.227 ) | 0.680065376 |  |  |
| Interleukin-16 levels | |  |  |  |  |  |
|  | MR Egger | 10 | 0.781 ( 0.353 - 1.728 ) | 0.55812913 | 0.146664035 | 0.4339448 |
|  | Weighted median | 10 | 1.047 ( 0.859 - 1.276 ) | 0.647665247 |  |  |
|  | Inverse variance weighted | 10 | 1.081 ( 0.910 - 1.284 ) | 0.374263232 | 0.156825372 |  |
|  | Simple mode | 10 | 1.057 ( 0.788 - 1.418 ) | 0.720121927 |  |  |
|  | Weighted mode | 10 | 1.046 ( 0.768 - 1.426 ) | 0.780289658 |  |  |
| Interleukin-4 levels | |  |  |  |  |  |
|  | MR Egger | 10 | 1.001 ( 0.632 - 1.585 ) | 0.998044184 | 0.312703862 | 0.850871837 |
|  | Weighted median | 10 | 1.009 ( 0.889 - 1.145 ) | 0.888652171 |  |  |
|  | Inverse variance weighted | 10 | 1.046 ( 0.951 - 1.151 ) | 0.356607666 | 0.4006823 |  |
|  | Simple mode | 10 | 0.979 ( 0.804 - 1.193 ) | 0.83944445 |  |  |
|  | Weighted mode | 10 | 1.008 ( 0.835 - 1.216 ) | 0.938205511 |  |  |
| Tumor necrosis factor alpha levels | |  |  |  |  |  |
|  | MR Egger | 10 | 1.398 ( 0.730 - 2.680 ) | 0.34202339 | 0.519043346 | 0.40246066 |
|  | Weighted median | 10 | 1.069 ( 0.880 - 1.299 ) | 0.49988645 |  |  |
|  | Inverse variance weighted | 10 | 1.050 ( 0.910 - 1.212 ) | 0.502233346 | 0.539642404 |  |
|  | Simple mode | 10 | 1.062 ( 0.780 - 1.444 ) | 0.712736713 |  |  |
|  | Weighted mode | 10 | 1.077 ( 0.796 - 1.457 ) | 0.642476831 |  |  |
| Interleukin-7 levels | |  |  |  |  |  |
|  | MR Egger | 10 | 1.424 ( 0.684 - 2.965 ) | 0.372443753 | 0.258780081 | 0.501962037 |
|  | Weighted median | 10 | 1.093 ( 0.902 - 1.325 ) | 0.365348122 |  |  |
|  | Inverse variance weighted | 10 | 1.102 ( 0.942 - 1.289 ) | 0.225848177 | 0.295880409 |  |
|  | Simple mode | 10 | 1.104 ( 0.830 - 1.467 ) | 0.514715701 |  |  |
|  | Weighted mode | 10 | 1.104 ( 0.819 - 1.487 ) | 0.533486439 |  |  |
| Interleukin-13 levels | |  |  |  |  |  |
|  | MR Egger | 10 | 1.292 ( 0.680 - 2.454 ) | 0.456979115 | 0.554609939 | 0.560882758 |
|  | Weighted median | 10 | 1.050 ( 0.879 - 1.255 ) | 0.592617654 |  |  |
|  | Inverse variance weighted | 10 | 1.064 ( 0.924 - 1.226 ) | 0.388715789 | 0.616058503 |  |
|  | Simple mode | 10 | 1.033 ( 0.799 - 1.336 ) | 0.810762624 |  |  |
|  | Weighted mode | 10 | 1.053 ( 0.821 - 1.350 ) | 0.693030257 |  |  |
| Interleukin-8 levels | |  |  |  |  |  |
|  | MR Egger | 10 | 0.799 ( 0.394 - 1.621 ) | 0.551544163 | 0.287113647 | 0.456463616 |
|  | Weighted median | 10 | 0.985 ( 0.812 - 1.195 ) | 0.878947773 |  |  |
|  | Inverse variance weighted | 10 | 1.053 ( 0.904 - 1.226 ) | 0.510674372 | 0.316305075 |  |
|  | Simple mode | 10 | 0.977 ( 0.723 - 1.320 ) | 0.88388098 |  |  |
|  | Weighted mode | 10 | 0.977 ( 0.761 - 1.255 ) | 0.860603231 |  |  |
| Macrophage colony stimulating factor levels | |  |  |  |  |  |
|  | MR Egger | 10 | 1.514 ( 0.697 - 3.290 ) | 0.325582803 | 0.843412823 | 0.270377407 |
|  | Weighted median | 10 | 0.975 ( 0.778 - 1.223 ) | 0.829203579 |  |  |
|  | Inverse variance weighted | 10 | 0.958 ( 0.808 - 1.136 ) | 0.622170229 | 0.783856419 |  |
|  | Simple mode | 10 | 1.095 ( 0.766 - 1.564 ) | 0.631360525 |  |  |
|  | Weighted mode | 10 | 1.110 ( 0.785 - 1.569 ) | 0.570649302 |  |  |
| Interleukin-17 levels | |  |  |  |  |  |
|  | MR Egger | 10 | 1.233 ( 0.748 - 2.032 ) | 0.435558204 | 0.225360244 | 0.479372495 |
|  | Weighted median | 10 | 0.999 ( 0.868 - 1.150 ) | 0.990191711 |  |  |
|  | Inverse variance weighted | 10 | 1.025 ( 0.921 - 1.141 ) | 0.651191141 | 0.253758464 |  |
|  | Simple mode | 10 | 1.016 ( 0.794 - 1.300 ) | 0.90333083 |  |  |
|  | Weighted mode | 10 | 1.033 ( 0.819 - 1.303 ) | 0.789689986 |  |  |
| Interleukin-1-receptor antagonist levels | |  |  |  |  |  |
|  | MR Egger | 10 | 1.487 ( 0.789 - 2.802 ) | 0.254594236 | 0.792320704 | 0.355950971 |
|  | Weighted median | 10 | 1.101 ( 0.925 - 1.311 ) | 0.279215724 |  |  |
|  | Inverse variance weighted | 10 | 1.092 ( 0.950 - 1.255 ) | 0.217335546 | 0.776447968 |  |
|  | Simple mode | 10 | 1.092 ( 0.842 - 1.416 ) | 0.523969059 |  |  |
|  | Weighted mode | 10 | 1.097 ( 0.853 - 1.411 ) | 0.487903526 |  |  |
| Platelet-derived growth factor BB levels | |  |  |  |  |  |
|  | MR Egger | 10 | 1.044 ( 0.656 - 1.660 ) | 0.860947369 | 0.286078511 | 0.837798574 |
|  | Weighted median | 10 | 1.062 ( 0.936 - 1.205 ) | 0.350005921 |  |  |
|  | Inverse variance weighted | 10 | 1.096 ( 0.995 - 1.207 ) | 0.063014623 | 0.370022434 |  |
|  | Simple mode | 10 | 1.044 ( 0.860 - 1.266 ) | 0.675044302 |  |  |
|  | Weighted mode | 10 | 1.052 ( 0.868 - 1.275 ) | 0.616276342 |  |  |
| Interleukin-18 levels | |  |  |  |  |  |
|  | MR Egger | 10 | 0.549 ( 0.186 - 1.626 ) | 0.310800241 | 0.002775881 | 0.298816869 |
|  | Weighted median | 10 | 1.140 ( 0.910 - 1.428 ) | 0.254436954 |  |  |
|  | Inverse variance weighted | 10 | 1.001 ( 0.786 - 1.276 ) | 0.993715518 | 0.001331777 |  |
|  | Simple mode | 10 | 1.229 ( 0.844 - 1.789 ) | 0.310150262 |  |  |
|  | Weighted mode | 10 | 1.248 ( 0.941 - 1.654 ) | 0.158139407 |  |  |
| Granulocyte-colony stimulating factor levels | |  |  |  |  |  |
|  | MR Egger | 10 | 1.253 ( 0.813 - 1.930 ) | 0.336275321 | 0.422867281 | 0.332174802 |
|  | Weighted median | 10 | 1.027 ( 0.906 - 1.164 ) | 0.676200819 |  |  |
|  | Inverse variance weighted | 10 | 1.004 ( 0.912 - 1.104 ) | 0.941919382 | 0.419989599 |  |
|  | Simple mode | 10 | 1.037 ( 0.865 - 1.242 ) | 0.704794558 |  |  |
|  | Weighted mode | 10 | 1.041 ( 0.869 - 1.246 ) | 0.67394722 |  |  |
| Monocyte chemoattractant protein-1 levels | |  |  |  |  |  |
|  | MR Egger | 10 | 0.985 ( 0.577 - 1.681 ) | 0.957322388 | 0.116443257 | 0.732548209 |
|  | Weighted median | 10 | 1.106 ( 0.964 - 1.269 ) | 0.15132032 |  |  |
|  | Inverse variance weighted | 10 | 1.082 ( 0.968 - 1.211 ) | 0.165927839 | 0.15945029 |  |
|  | Simple mode | 10 | 1.081 ( 0.829 - 1.409 ) | 0.57912643 |  |  |
|  | Weighted mode | 10 | 1.120 ( 0.862 - 1.454 ) | 0.418621056 |  |  |
| Fibroblast growth factor basic levels | |  |  |  |  |  |
|  | MR Egger | 10 | 1.302 ( 0.840 - 2.018 ) | 0.271748757 | 0.468893735 | 0.29337574 |
|  | Weighted median | 10 | 0.984 ( 0.860 - 1.125 ) | 0.810483934 |  |  |
|  | Inverse variance weighted | 10 | 1.019 ( 0.925 - 1.122 ) | 0.704952093 | 0.445654727 |  |
|  | Simple mode | 10 | 0.969 ( 0.777 - 1.207 ) | 0.783575461 |  |  |
|  | Weighted mode | 10 | 0.969 ( 0.776 - 1.209 ) | 0.785047985 |  |  |
| Monokine induced by gamma interferon levels | |  |  |  |  |  |
|  | MR Egger | 10 | 0.618 ( 0.330 - 1.158 ) | 0.171698715 | 0.464726293 | 0.127744637 |
|  | Weighted median | 10 | 1.070 ( 0.886 - 1.293 ) | 0.481328534 |  |  |
|  | Inverse variance weighted | 10 | 1.051 ( 0.905 - 1.222 ) | 0.514044109 | 0.306142504 |  |
|  | Simple mode | 10 | 1.079 ( 0.797 - 1.460 ) | 0.635459581 |  |  |
|  | Weighted mode | 10 | 1.063 ( 0.817 - 1.383 ) | 0.661542788 |  |  |
| Hepatocyte growth factor levels | |  |  |  |  |  |
|  | MR Egger | 10 | 1.112 ( 0.640 - 1.931 ) | 0.715860055 | 0.087938916 | 0.996654306 |
|  | Weighted median | 10 | 1.093 ( 0.952 - 1.256 ) | 0.207239981 |  |  |
|  | Inverse variance weighted | 10 | 1.111 ( 0.990 - 1.246 ) | 0.072753667 | 0.130714399 |  |
|  | Simple mode | 10 | 1.067 ( 0.807 - 1.411 ) | 0.65809209 |  |  |
|  | Weighted mode | 10 | 1.258 ( 0.961 - 1.648 ) | 0.129175796 |  |  |
| Tumor necrosis factor beta levels | |  |  |  |  |  |
|  | MR Egger | 9 | 1.063 ( 0.370 - 3.051 ) | 0.913204482 | 0.878810956 | 0.819039539 |
|  | Weighted median | 9 | 0.908 ( 0.684 - 1.205 ) | 0.5035637 |  |  |
|  | Inverse variance weighted | 9 | 0.938 ( 0.751 - 1.172 ) | 0.572118504 | 0.926427604 |  |
|  | Simple mode | 9 | 0.921 ( 0.600 - 1.412 ) | 0.714884113 |  |  |
|  | Weighted mode | 9 | 0.908 ( 0.599 - 1.377 ) | 0.661409552 |  |  |
| Vascular endothelial growth factor levels | |  |  |  |  |  |
|  | MR Egger | 10 | 0.959 ( 0.599 - 1.535 ) | 0.865288403 | 0.37962825 | 0.545379437 |
|  | Weighted median | 10 | 1.062 ( 0.926 - 1.218 ) | 0.390985135 |  |  |
|  | Inverse variance weighted | 10 | 1.112 ( 1.006 - 1.229 ) | 0.038346458 | 0.437296305 |  |
|  | Simple mode | 10 | 1.016 ( 0.823 - 1.254 ) | 0.887994344 |  |  |
|  | Weighted mode | 10 | 1.046 ( 0.850 - 1.288 ) | 0.679257105 |  |  |
| Growth-regulated protein alpha levels | |  |  |  |  |  |
|  | MR Egger | 10 | 0.987 ( 0.518 - 1.879 ) | 0.968781229 | 0.591351941 | 0.896382066 |
|  | Weighted median | 10 | 1.104 ( 0.914 - 1.334 ) | 0.304788714 |  |  |
|  | Inverse variance weighted | 10 | 1.030 ( 0.894 - 1.188 ) | 0.681009904 | 0.687102479 |  |
|  | Simple mode | 10 | 1.131 ( 0.839 - 1.524 ) | 0.44145562 |  |  |
|  | Weighted mode | 10 | 1.123 ( 0.857 - 1.472 ) | 0.423312189 |  |  |
| Interleukin-6 levels | |  |  |  |  |  |
|  | MR Egger | 10 | 1.253 ( 0.820 - 1.913 ) | 0.327604769 | 0.629746673 | 0.494730978 |
|  | Weighted median | 10 | 1.053 ( 0.931 - 1.190 ) | 0.411650345 |  |  |
|  | Inverse variance weighted | 10 | 1.077 ( 0.981 - 1.183 ) | 0.117803047 | 0.671652862 |  |
|  | Simple mode | 10 | 1.033 ( 0.850 - 1.256 ) | 0.751356212 |  |  |
|  | Weighted mode | 10 | 1.048 ( 0.875 - 1.256 ) | 0.620225305 |  |  |
| Stem cell factor levels | |  |  |  |  |  |
|  | MR Egger | 10 | 0.894 ( 0.587 - 1.361 ) | 0.615631318 | 0.648559461 | 0.641334367 |
|  | Weighted median | 10 | 1.022 ( 0.907 - 1.151 ) | 0.724783802 |  |  |
|  | Inverse variance weighted | 10 | 0.989 ( 0.902 - 1.085 ) | 0.820005452 | 0.717465939 |  |
|  | Simple mode | 10 | 1.020 ( 0.856 - 1.215 ) | 0.829762174 |  |  |
|  | Weighted mode | 10 | 1.017 ( 0.848 - 1.219 ) | 0.859585734 |  |  |
| Eotaxin levels | |  |  |  |  |  |
|  | MR Egger | 10 | 1.065 ( 0.697 - 1.626 ) | 0.779605844 | 0.841675684 | 0.664910148 |
|  | Weighted median | 10 | 0.988 ( 0.880 - 1.109 ) | 0.832035933 |  |  |
|  | Inverse variance weighted | 10 | 0.968 ( 0.882 - 1.063 ) | 0.498850012 | 0.885420804 |  |
|  | Simple mode | 10 | 1.003 ( 0.835 - 1.205 ) | 0.971939146 |  |  |
|  | Weighted mode | 10 | 1.008 ( 0.837 - 1.215 ) | 0.932959905 |  |  |
| Interleukin-2 levels | |  |  |  |  |  |
|  | MR Egger | 10 | 1.992 ( 0.777 - 5.108 ) | 0.189332805 | 0.031192961 | 0.354844871 |
|  | Weighted median | 10 | 1.192 ( 0.966 - 1.471 ) | 0.101468286 |  |  |
|  | Inverse variance weighted | 10 | 1.257 ( 1.022 - 1.546 ) | 0.030044711 | 0.025754611 |  |
|  | Simple mode | 10 | 1.185 ( 0.832 - 1.689 ) | 0.371306272 |  |  |
|  | Weighted mode | 10 | 1.122 ( 0.848 - 1.484 ) | 0.441194946 |  |  |
| Macrophage inflammatory protein 1a levels | |  |  |  |  |  |
|  | MR Egger | 10 | 1.708 ( 0.879 - 3.319 ) | 0.152941912 | 0.386638514 | 0.268696511 |
|  | Weighted median | 10 | 1.130 ( 0.932 - 1.371 ) | 0.21369038 |  |  |
|  | Inverse variance weighted | 10 | 1.153 ( 0.993 - 1.339 ) | 0.062318074 | 0.350852998 |  |
|  | Simple mode | 10 | 1.106 ( 0.811 - 1.508 ) | 0.541765496 |  |  |
|  | Weighted mode | 10 | 1.143 ( 0.823 - 1.587 ) | 0.44638947 |  |  |
| Interferon gamma levels | |  |  |  |  |  |
|  | MR Egger | 10 | 0.990 ( 0.613 - 1.597 ) | 0.967566032 | 0.290843983 | 0.960172059 |
|  | Weighted median | 10 | 0.942 ( 0.819 - 1.084 ) | 0.40582469 |  |  |
|  | Inverse variance weighted | 10 | 0.978 ( 0.885 - 1.080 ) | 0.657661585 | 0.379722929 |  |
|  | Simple mode | 10 | 0.873 ( 0.686 - 1.109 ) | 0.29419281 |  |  |
|  | Weighted mode | 10 | 0.886 ( 0.686 - 1.144 ) | 0.376590439 |  |  |
| Interleukin-9 levels | |  |  |  |  |  |
|  | MR Egger | 10 | 1.507 ( 0.661 - 3.437 ) | 0.358287255 | 0.095488703 | 0.269561535 |
|  | Weighted median | 10 | 0.987 ( 0.818 - 1.190 ) | 0.8884601 |  |  |
|  | Inverse variance weighted | 10 | 0.926 ( 0.769 - 1.115 ) | 0.417340815 | 0.069313179 |  |
|  | Simple mode | 10 | 1.008 ( 0.757 - 1.342 ) | 0.959896348 |  |  |
|  | Weighted mode | 10 | 1.016 ( 0.777 - 1.327 ) | 0.912672553 |  |  |
| Interleukin-5 levels | |  |  |  |  |  |
|  | MR Egger | 10 | 1.548 ( 0.801 - 2.990 ) | 0.22967327 | 0.515836396 | 0.22302243 |
|  | Weighted median | 10 | 1.006 ( 0.827 - 1.224 ) | 0.950036509 |  |  |
|  | Inverse variance weighted | 10 | 1.004 ( 0.868 - 1.161 ) | 0.957217999 | 0.442896077 |  |
|  | Simple mode | 10 | 0.987 ( 0.729 - 1.337 ) | 0.93583828 |  |  |
|  | Weighted mode | 10 | 0.994 ( 0.755 - 1.309 ) | 0.966423592 |  |  |
| Monocyte chemoattractant protein-3 levels | |  |  |  |  |  |
|  | MR Egger | 10 | 1.035 ( 0.315 - 3.401 ) | 0.956373987 | 0.408744721 | 0.99217324 |
|  | Weighted median | 10 | 1.034 ( 0.737 - 1.450 ) | 0.847454592 |  |  |
|  | Inverse variance weighted | 10 | 1.041 ( 0.805 - 1.346 ) | 0.758963656 | 0.508424618 |  |
|  | Simple mode | 10 | 0.993 ( 0.551 - 1.788 ) | 0.981382132 |  |  |
|  | Weighted mode | 10 | 1.012 ( 0.562 - 1.824 ) | 0.968433169 |  |  |
| RANTES levels | |  |  |  |  |  |
|  | MR Egger | 10 | 0.550 ( 0.286 - 1.056 ) | 0.110176222 | 0.732124613 | 0.077288261 |
|  | Weighted median | 10 | 1.018 ( 0.842 - 1.232 ) | 0.850523114 |  |  |
|  | Inverse variance weighted | 10 | 1.062 ( 0.917 - 1.230 ) | 0.422500493 | 0.406333901 |  |
|  | Simple mode | 10 | 1.000 ( 0.765 - 1.306 ) | 0.997587675 |  |  |
|  | Weighted mode | 10 | 0.991 ( 0.763 - 1.286 ) | 0.946323102 |  |  |
| Macrophage inflammatory protein 1b levels | |  |  |  |  |  |
|  | MR Egger | 10 | 0.855 ( 0.476 - 1.537 ) | 0.615289888 | 0.0500183 | 0.565428611 |
|  | Weighted median | 10 | 1.038 ( 0.906 - 1.190 ) | 0.587059579 |  |  |
|  | Inverse variance weighted | 10 | 1.019 ( 0.899 - 1.154 ) | 0.770573648 | 0.062764932 |  |
|  | Simple mode | 10 | 1.070 ( 0.885 - 1.293 ) | 0.504926312 |  |  |
|  | Weighted mode | 10 | 1.070 ( 0.896 - 1.276 ) | 0.474929695 |  |  |
| Stromal-cell-derived factor 1 alpha levels | |  |  |  |  |  |
|  | MR Egger | 10 | 1.410 ( 0.840 - 2.366 ) | 0.23002144 | 0.181323472 | 0.256064675 |
|  | Weighted median | 10 | 1.012 ( 0.878 - 1.165 ) | 0.872641146 |  |  |
|  | Inverse variance weighted | 10 | 1.028 ( 0.915 - 1.156 ) | 0.638755194 | 0.141141952 |  |
|  | Simple mode | 10 | 0.958 ( 0.730 - 1.256 ) | 0.762947157 |  |  |
|  | Weighted mode | 10 | 0.975 ( 0.745 - 1.277 ) | 0.860460945 |  |  |
| Interferon gamma-induced protein 10 levels | |  |  |  |  |  |
|  | MR Egger | 10 | 0.895 ( 0.477 - 1.680 ) | 0.739625292 | 0.436557513 | 0.510516456 |
|  | Weighted median | 10 | 1.145 ( 0.949 - 1.382 ) | 0.158476547 |  |  |
|  | Inverse variance weighted | 10 | 1.111 ( 0.967 - 1.276 ) | 0.137280261 | 0.490220412 |  |
|  | Simple mode | 10 | 1.152 ( 0.854 - 1.554 ) | 0.379311227 |  |  |
|  | Weighted mode | 10 | 1.157 ( 0.872 - 1.535 ) | 0.337515686 |  |  |
| Interleukin-2 receptor antagonist levels | |  |  |  |  |  |
|  | MR Egger | 10 | 1.025 ( 0.525 - 1.999 ) | 0.944600801 | 0.342097074 | 0.78656928 |
|  | Weighted median | 10 | 1.001 ( 0.828 - 1.211 ) | 0.991015835 |  |  |
|  | Inverse variance weighted | 10 | 0.934 ( 0.812 - 1.073 ) | 0.334716991 | 0.42895423 |  |
|  | Simple mode | 10 | 1.031 ( 0.735 - 1.447 ) | 0.862857029 |  |  |
|  | Weighted mode | 10 | 1.037 ( 0.774 - 1.389 ) | 0.813599098 |  |  |
| Interleukin-1-beta levels | |  |  |  |  |  |
|  | MR Egger | 10 | 1.262 ( 0.763 - 2.090 ) | 0.391354219 | 0.720040434 | 0.417258049 |
|  | Weighted median | 10 | 1.005 ( 0.874 - 1.155 ) | 0.94693749 |  |  |
|  | Inverse variance weighted | 10 | 1.019 ( 0.912 - 1.138 ) | 0.744387372 | 0.732136325 |  |
|  | Simple mode | 10 | 1.045 ( 0.859 - 1.272 ) | 0.670363798 |  |  |
|  | Weighted mode | 10 | 1.033 ( 0.844 - 1.264 ) | 0.759503219 |  |  |
| Interleukin-12p70 levels | |  |  |  |  |  |
|  | MR Egger | 10 | 1.044 ( 0.659 - 1.653 ) | 0.859732329 | 0.29928717 | 0.885927774 |
|  | Weighted median | 10 | 1.046 ( 0.920 - 1.191 ) | 0.490677286 |  |  |
|  | Inverse variance weighted | 10 | 1.080 ( 0.981 - 1.188 ) | 0.116186087 | 0.387275382 |  |
|  | Simple mode | 10 | 0.992 ( 0.796 - 1.236 ) | 0.945231716 |  |  |
|  | Weighted mode | 10 | 0.990 ( 0.788 - 1.244 ) | 0.934207538 |  |  |
| Macrophage Migration Inhibitory Factor levels | |  |  |  |  |  |
|  | MR Egger | 10 | 0.643 ( 0.338 - 1.226 ) | 0.217006744 | 0.555163903 | 0.195753128 |
|  | Weighted median | 10 | 0.974 ( 0.805 - 1.178 ) | 0.785695176 |  |  |
|  | Inverse variance weighted | 10 | 1.012 ( 0.878 - 1.167 ) | 0.86779409 | 0.453874939 |  |
|  | Simple mode | 10 | 0.893 ( 0.675 - 1.181 ) | 0.448567572 |  |  |
|  | Weighted mode | 10 | 0.931 ( 0.705 - 1.229 ) | 0.625782258 |  |  |
| TRAIL levels | |  |  |  |  |  |
|  | MR Egger | 10 | 1.152 ( 0.564 - 2.354 ) | 0.7074819 | 0.00344764 | 0.699387004 |
|  | Weighted median | 10 | 0.937 ( 0.806 - 1.088 ) | 0.3897554 |  |  |
|  | Inverse variance weighted | 10 | 0.999 ( 0.860 - 1.161 ) | 0.994006938 | 0.005366998 |  |
|  | Simple mode | 10 | 0.866 ( 0.622 - 1.208 ) | 0.419657414 |  |  |
|  | Weighted mode | 10 | 0.869 ( 0.623 - 1.212 ) | 0.429886998 |  |  |
| CTACK levels | |  |  |  |  |  |
|  | MR Egger | 10 | 1.377 ( 0.732 - 2.593 ) | 0.350351535 | 0.49215981 | 0.512034534 |
|  | Weighted median | 10 | 0.977 ( 0.814 - 1.173 ) | 0.806537035 |  |  |
|  | Inverse variance weighted | 10 | 1.110 ( 0.965 - 1.276 ) | 0.143945758 | 0.545282213 |  |
|  | Simple mode | 10 | 0.966 ( 0.739 - 1.262 ) | 0.806040665 |  |  |
|  | Weighted mode | 10 | 0.965 ( 0.732 - 1.270 ) | 0.803000853 |  |  |
| Stem cell growth factor beta levels | |  |  |  |  |  |
|  | MR Egger | 10 | 0.634 ( 0.338 - 1.188 ) | 0.19248829 | 0.442561052 | 0.116866417 |
|  | Weighted median | 10 | 1.094 ( 0.897 - 1.335 ) | 0.372828276 |  |  |
|  | Inverse variance weighted | 10 | 1.098 ( 0.942 - 1.280 ) | 0.23187194 | 0.275931375 |  |
|  | Simple mode | 10 | 0.980 ( 0.674 - 1.424 ) | 0.918542445 |  |  |
|  | Weighted mode | 10 | 1.035 ( 0.746 - 1.435 ) | 0.841708405 |  |  |
| beta-nerve growth factor levels | |  |  |  |  |  |
|  | MR Egger | 10 | 0.565 ( 0.296 - 1.075 ) | 0.120166495 | 0.511065823 | 0.098187281 |
|  | Weighted median | 10 | 1.004 ( 0.828 - 1.217 ) | 0.968594408 |  |  |
|  | Inverse variance weighted | 10 | 1.029 ( 0.881 - 1.201 ) | 0.71960307 | 0.293846804 |  |
|  | Simple mode | 10 | 0.962 ( 0.736 - 1.258 ) | 0.782411289 |  |  |
|  | Weighted mode | 10 | 0.992 ( 0.764 - 1.288 ) | 0.953120709 |  |  |

Abbreviations: SNP, single nucleotide polymorphism; OR, odds ratio; CI, confidence interval.
