# Supplementary material for: The Association Between Genetically Predicted Systemic Inflammatory Regulators and Polycystic Ovary Syndrome: A Mendelian Randomization Study
Source: Front Endocrinol (Lausanne). 2021 Sep 27;12:731569. doi: 10.3389/fendo.2021.731569 (PMC8503255; doi:10.3389/fendo.2021.731569)
Supplement: Supplementary file 1 [file DataSheet_1.zip › Data Sheet 1/supplementary materials/Supplementary Table S7.docx]

**Supplementary Table S7.** Differences in the expression level of associated systemic inflammatory regulators between PCOS group and control group.

| **Systemic Inflammatory Regulators (pg/ml)** | **Control Group** | **PCOS Group** | **P** | **OR (95% CI)** | **P_OR_** |
| --- | --- | --- | --- | --- | --- |
| IL-2 | 11.38±19.52 | 13.08±21.51 | 0.87 | 1.003(0.967-1.041) | 0.87 |
| IL-4 | 2.85±0.71 | 2.75±0.52 | 0.27 | 0.752(0.227-2.493) | 0.64 |
| IL-17 | 50.87±20.38 | 37.31±17.47 | 0.56 | 0.960(0.917-1.004) | 0.08 |
| SCGFb | 81.47±53.12 | 56.57±45.63 | 0.55 | 0.989(0.973-1.0105) | 0.19 |
| SDF1a | 2656.1±1641.18 | 2687.83±679.53 | 0.26 | 1.000(0.999-1.001) | 0.94 |
| VEGF | 269.94±99.375 | 290.09±128.62 | 0.65 | 1.002(0.995-1.008) | 0.64 |

OR, odds ratio; CI, confidence interval; IL-2, interleukin-2; IL-4, interleukin-4; IL-17, interleukin-17; SCGFb, stem cell growth factor beta; SDF1a, stromal-cell-derived factor 1 alpha; VEGF, vascular endothelial growth factor. Data were shown as means±standard deviations.
