# Supplementary material for: The Association Between Genetically Predicted Systemic Inflammatory Regulators and Polycystic Ovary Syndrome: A Mendelian Randomization Study
Source: Front Endocrinol (Lausanne). 2021 Sep 27;12:731569. doi: 10.3389/fendo.2021.731569 (PMC8503255; doi:10.3389/fendo.2021.731569)
Supplement: Supplementary file 1 [file DataSheet_1.zip › Data Sheet 1/supplementary materials/Supplementary_Material.docx]

Supplementary Material

# Supplementary Tables

**Supplementary Table S1.** Characteristics of the genetic instrument variables for the systemic inflammatory regulators in the Mendelian randomization study at the genome-wide significance level (P < 5 × 10^–8^).

**Supplementary Table S2.** Characteristics of the genetic instrument variables for the systemic inflammatory regulators in the Mendelian randomization study at P < 5 × 10^–6^ significance level.

**Supplementary Table S3.** SNPs used as instrument variables for PCOS.

**Supplementary Table S4.** MR analysis of 17 systemic inflammatory regulators and PCOS risk.

**Supplementary Table S5.** MR analysis of 41 systemic inflammatory regulators and PCOS risk.

**Supplementary Table S6.** MR analysis of the association between PCOS and 41 systemic inflammatory regulators.

**Supplementary Table S7.** Differences in the expression level of associated systemic inflammatory regulators between PCOS group and control group.
